# Therapy-induced modulation of tumor vasculature and oxygenation in a murine glioblastoma model quantified by deep learning-based feature extraction

**DOI:** 10.1038/s41598-024-52268-0

**Published:** 2024-01-23

**Authors:** Nadine Bauer, Daniel Beckmann, Dirk Reinhardt, Nicole Frost, Stefanie Bobe, Raghu Erapaneedi, Benjamin Risse, Friedemann Kiefer

**Affiliations:** 1 European Institute for Molecular Imaging (EIMI), Multiscale Imaging Centre (MIC), University of Münster, Röntgenstr. 16, 48149 Münster, Germany; 2 Max Planck Institute for Molecular Biomedicine, Röntgenstr. 20, 48149 Münster, Germany; 3 Institute for Geoinformatics, University of Münster, Heisenbergstr. 2, 48149 Münster, Germany; 4 Institute for Computer Science, University of Münster, Einsteinstraße 62, 48149 Münster, Germany; 5 Gerhard Domagk Institute of Pathology, University Hospital Münster, Domagkstr. 15, 48149 Münster, Germany

**Keywords:** Light-sheet microscopy, Tumour angiogenesis, Computational science, Fluorescence imaging, Cancer therapy

## Abstract

Glioblastoma presents characteristically with an exuberant, poorly functional vasculature that causes malperfusion, hypoxia and necrosis. Despite limited clinical efficacy, anti-angiogenesis resulting in vascular normalization remains a promising therapeutic approach. Yet, fundamental questions concerning anti-angiogenic therapy remain unanswered, partly due to the scale and resolution gap between microscopy and clinical imaging and a lack of quantitative data readouts. To what extend does treatment lead to vessel regression or vessel normalization and does it ameliorate or aggravate hypoxia? Clearly, a better understanding of the underlying mechanisms would greatly benefit the development of desperately needed improved treatment regimens. Here, using orthotopic transplantation of Gli36 cells, a widely used murine glioma model, we present a mesoscopic approach based on light sheet fluorescence microscopic imaging of wholemount stained tumors. Deep learning-based segmentation followed by automated feature extraction allowed quantitative analyses of the entire tumor vasculature and oxygenation statuses. Unexpectedly in this model, the response to both cytotoxic and anti-angiogenic therapy was dominated by vessel normalization with little evidence for vessel regression. Equally surprising, only cytotoxic therapy resulted in a significant alleviation of hypoxia. Taken together, we provide and evaluate a quantitative workflow that addresses some of the most urgent mechanistic questions in anti-angiogenic therapy.

## Introduction

Glioblastoma multiforme (GBM), the most aggressive malignant primary tumor of the central nervous system, is characterized by high levels of pro-angiogenic growth factors that induce microvascular proliferation and development of dysfunctional, structurally abnormal vessels^1^. Resulting poor perfusion leads to formation of hypoxic areas, a hallmark of this tumor type, which favor malignant progression and contribute to therapy resistance^2^. Standard multimodality treatment, comprising surgery plus the alkylating cytotoxin temozolomide (TMZ) and radiotherapy (chemoradiation), only poorly extends the overall low survival rate^3,4^. Tumor-induced angiogenesis as an essential prerequisite for tumor growth, and consequently anti-angiogenesis as a treatment modality, was first proposed by Folkman^5^ and became feasible after identification of the endothelial-selective growth factor vascular endothelial growth factor A (VEGF-A)^6,7^. Notoriously high VEGF-A expression in GBM raised high hopes for anti-angiogenic therapy (AAT), however treatment of recurrent GBM by combination of chemoradiation and the monoclonal, humanized VEGF-A binding antibody bevacizumab (BVZ) failed to prolong overall survival^6–8^. Like for other tumor entities also in GBM, counterintuitively, the combination of AAT and chemotherapy was more effective than monotherapy. This is at odds with the initial model of AAT-mediated blood vessel regression and tumor dormancy and resulted in the development of a modified concept, vascular normalization. Central to vascular normalization is the neutralization of excessive tumor-derived pro-angiogenic activity, resulting in vessel stabilization and improved perfusion^9^. However, the relative contribution of vascular regression and vessel stabilization to vascular normalization remains unclear. Comprehensive data, whether BVZ induces vessel regression, promotes hypoxia formation and therefore malignant progression or if it acts preferentially via improved perfusion and alleviates tumor hypoxia, are lacking^10^. The situation is even more complex for additional AAT targets. A prime example is cilengitide (CIL), a cyclic RGD-peptide designed to interfere with αvβ3- and αvβ5-integrin-mediated angiogenesis and to suppress tumor growth^11^. Unfortunately, treatment with highly dosed CIL (hd CIL; also in combination with TMZ) failed to improve patient survival^12^ and showed only a poor anti-angiogenic effect in several clinical trials^13,14^. Most interestingly, even adverse effects were observed in tumors treated with low dosed CIL (ld CIL), which induced VEGF-A mediated vascular promotion and hyper-vascularization as an effect of compensatory VEGF-receptor 2 (VEGFR-2) up-regulation^15,16^.

To achieve a higher level in understanding AAT and vascular normalization mechanistically, novel, quantitative approaches to assess tumor vascular networks that complement conventional histological and cell biological analysis, are required^17,18^. Over the last decades, the transition from analyzing tissue sections to tissue volumes became possible through the introduction of disruptive technologies in image acquisition and evaluation^19,20^. Light sheet fluorescence microscopes and the development of bespoke wholemount staining and tissue clearing protocols now allow the rapid recording of cubic centimetre-sized image stacks representing entire organs at cellular resolution^21–24^. Hence, light sheet fluorescence microscopy (LSFM) allows collection of a high amount of data points within a volume of one specimen, which drastically increases the statistical basis when compared to datasets derived from sections. Therefore, LSFM is optimally suited to comprehend the tumor microenvironment and vascular networks of unsectioned tissue volumes. Yet, analysis of image volume information becomes substantially more difficult with growing size and resolution, making manual quantitation infeasible. Therefore, processing and evaluating LSFM datasets of hundreds of gigabytes requires automated high-throughput algorithms and workflows for the extraction and quantification of biological features. In contrast to 2D images, analyzing image volumes bears additional computational complexity and challenges. Recently, several methods for automatic feature extraction of vascular structures from large 3D image volumes, based on segmentation and network structure identification, were introduced^25–30^. Segmentation by classical methods usually requires manual feature engineering and parameter adjustments^23,30,31^, to which supervised, automated machine learning offers an alternative ^27,28,32,33^. Due to high variability of LSFM data, manual feature engineering often fails, hence machine learning has gained increasing popularity^34^. Once segmentations are computed, the underlying network structure can be quantified by classical approaches such as skeletonization^35^, which benefit from explicit parameterizations allowing to extract statistical yet interpretable characteristics.

Here, we report an extensive computational analysis of the architectural features of the vascular network and the associated hypoxia microenvironment in orthotopically transplanted glioblastoma xenografts. Pivotal to the approach is a quantitative description of the vascular bed of the entire tumor, while at the same time the effects of different treatment regimens comprising AAT mono- or AAT/cytotoxic combination therapy are assessed. To visualize sites of tumor hypoxia, a modified, genetically encoded UnaG-based hypoxia-sensor (HRE-dUnaG-3ALFA, Supplementary Figure S1) was utilized^36,37^. Newly inserted ALFA-epitope tags^38^ in combination with a hyperhydration step for improved tissue permeabilization permitted detection of intratumoral hypoxia by wholemount staining. The workflow for the analysis of entire tumor volumes of LSFM image stacks included machine learning-based segmentation of the vascular network by applying a customized training procedure to target large and abnormal vessels, followed by skeletonization and feature extraction of the obtained graph representations and concluded by identification of the differentially oxygenated tumor regions. Using this pipeline to quantify the vasculature of brain tumors, unexpectedly revealed that the response to both cytotoxic and anti-angiogenic therapy was dominated by vessel normalization with little evidence for vessel regression. Equally surprising, only cytotoxic therapy resulted in a significant alleviation of hypoxia. Therefore, we are here providing a model and roadmap to dissect the vascular modulating mechanisms of AAT and their influence on tumor oxygenation patterns in preclinical tumor models.

## Results

### Comprehensive visualization of tumor hypoxia in a murine glioblastoma model

The vasculature of GBM is structurally highly abnormal, containing convoluted large diameter vessels, leading to poor perfusion and hypoxia. We aimed to analyze the impact of vascular normalization on tumor vessel structure and hypoxia in the widely used Gli36 mouse glioma model. For intratumoral hypoxia, we applied our recently published genetically encoded hypoxia sensor HRE-dUnaG, which labels hypoxic cells in a HIF (hypoxia-inducible factor)-dependent fashion^36^. This sensor, designed for live cell and intravital microscopy, is not applicable for LSFM analysis of organically cleared samples, where fluorescence of the reporter protein UnaG-fluorescence is lost. To asses tumor hypoxia in organically cleared brains, we fused three copies of the ALFA-epitope tag^38^ to UnaG, resulting in the modified sensor HRE-dUnaG-3ALFA. The tag was detected by an ATTO542-directly labelled bacterially-expressed α-ALFA-nanobody and specificity of the approach was demonstrated in transfected Gli36 cells (Supplementary Figure S1). Also in confocal images of cyrosections from constitutively dUnaG-3ALFA expressing intracranial tumors, the UnaG fluorescence and the immunostained AFLA-tag colocalized (Supplementary Figure S2a). In a first step, we approached the development of a wholemount staining protocol for entire brains by analysis of 1 mm vibratome sections that were fully penetrated by the nanobodies as demonstrated by LSFM. As expected, UnaG fluorescence was lost during optical clearing and refractory index-matching with BABB, while the ATTO-labelled α-ALFA nanobodies were readily detectable (Supplementary Figure S2b).

**Figure 1:**
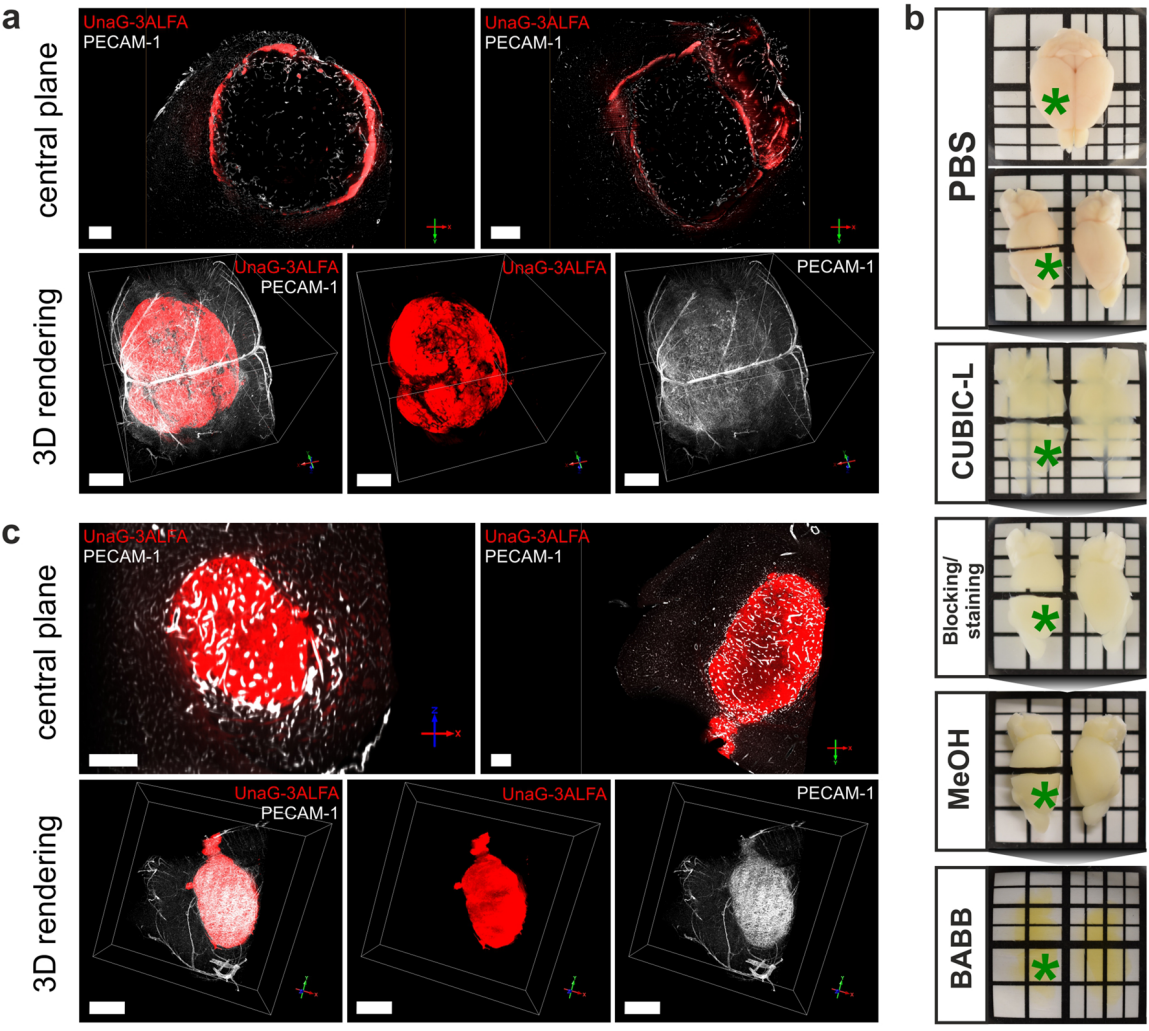
Wholemount staining of constitutively dUnaG-3ALFA-expressing intracranial tumors (Gli36). Prior to dissection, labelled α-PECAM-1 antibodies (cerebral and tumor vasculature: white) were intravenously injected into tumor bearing SCID mice, while tumors were wholemount stained for dUnaG-3ALFA expression levels *post-mortem*. (**a**) Triton X-100 based permeabilization prior to immunostaining with α-ALFA targeting fluorophore-labelled nanobodies (red) analyzed by light sheet fluorescence microscopy (scale bars: 400 µm (central plane); bars correspond to 1 mm in x/y plane (3D rendering)).(**b**) Stereomicroscopic view of a mouse brain after dissection, splitting of the hemispheres and division of the right hemisphere in two pieces. Subsequent panels show the same specimen after incubation in CUBIC-L, blocking and staining, dehydration and clearing in BABB (green asterisk indicates site of tumor). (**c**) Light sheet microscopic visualization of tumor wholemount stained following the procedure outlined in (**b**) (scale bars: 400 µm (central plane); bars correspond to 1 mm in x/y plane (3D rendering)).

For wholemount immunostaining of an entire brain hemisphere, harbouring a constitutively dUnaG-3ALFA expressing tumor, we applied our previously developed protocol based on Triton X-100 permeabilization. However, LSFM analysis revealed insufficient penetration of the GBM tumor by α-ALFA nanobodies, which only decorated a surface layer of approx. 200 µm, but failed to reach deeper tumor tissue (Fig. 1a). Albeit not stained due to absence of the cognate epitope, we conclude that the surrounding brain tissue was readily penetrated by the α-ALFA-nanobodies, which then were unable to enter the significantly denser tumor tissue. Aiming to develop an improved wholemount staining protocol for the dense glioma tissue, we introduced incubation in the reagent CUBIC-L for efficient permeabilization, delipidation and decolorization before proceeding to nanobody staining^39^. CUBIC-L, which contains the tertiary amine *N*,*N*-Bis(2-hydroxyethyl)butylamine, efficiently delipidates and decolorizes lipid-rich samples like human brain, while preserving proteins and their antigenicity. After perfusion fixation, dissection and sagittal separation of the tumor bearing and contralateral hemispheres, samples were incubated in CUBIC-L, where they swelled due to the reagent’s hyper-hydrating properties (Fig. 1b, tumor location: green asterisk). Blocking of non-specific epitopes was followed by immunostaining, applying our original wholemount staining protocol, and finally optical clearing in BABB. Applying this modified protocol to intracranial tumors that constitutively expressed dUnaG-3ALFA, resulted in satisfactory staining of the reporter throughout the entire volume (Fig. 1c).

**Figure 2:**
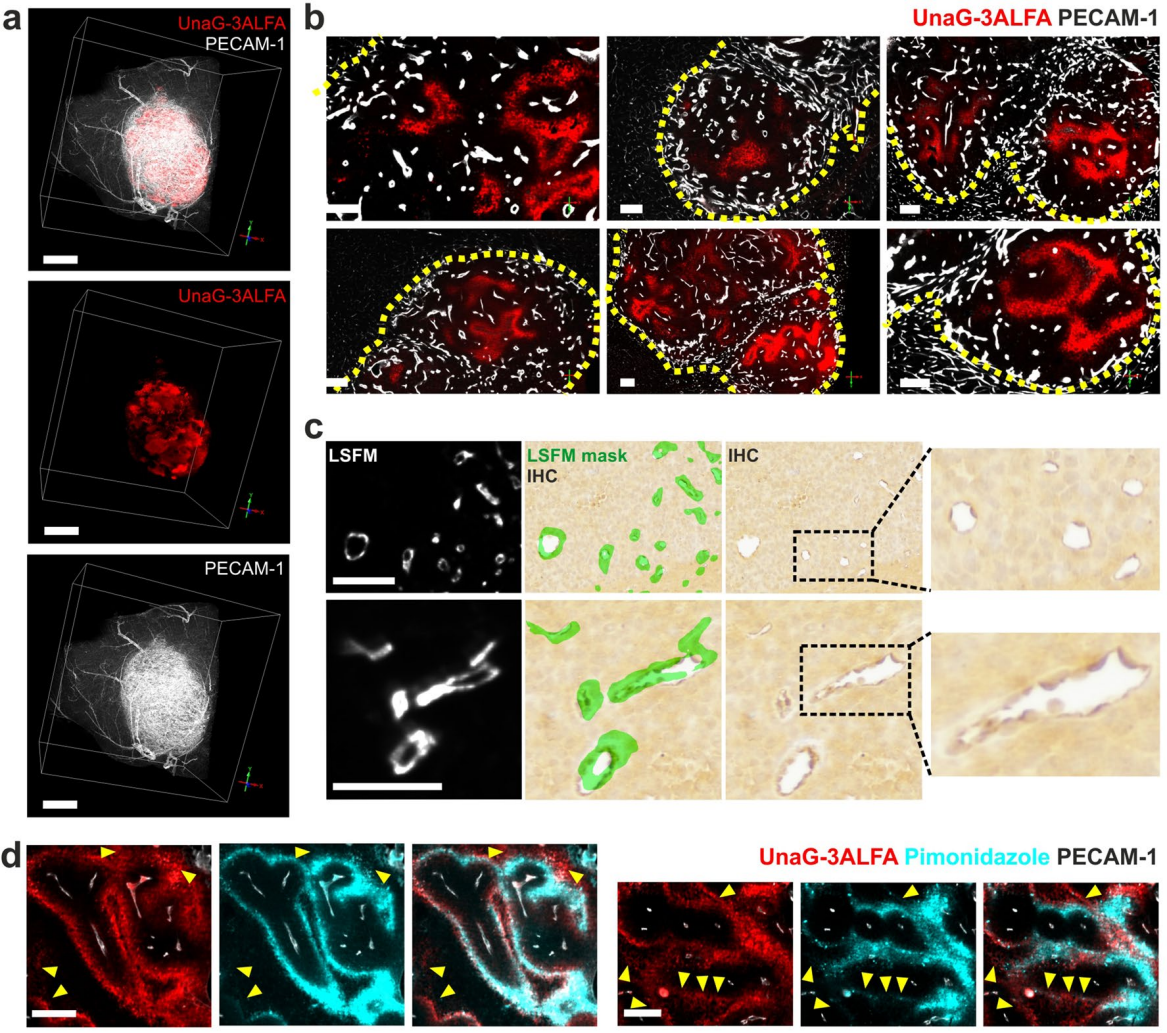
Visualization of hypoxic tumor regions. An intracranial tumor (Gli36) expressing dUnaG-3ALFA under the control of a hypoxia-inducible (HRE) synthetic promotor, was intravenously (i.v.) labelled by α-PECAM-1 antibodies (cerebral and tumor vasculature: white) and immunostained with α-ALFA nanobodies (hypoxic tumor regions: red) (**a**) Volume rendering of the tumor imaged by light sheet fluorescence microscopy (LSFM; bars correspond to 1 mm in x/y plane). (**b**) Visualization of hypoxic regions in individual optical sections across the tumor shown in (**a**), tumor margins are indicated by yellow dotted lines (scale bars: 200 µm). (**c**) Correlation of tumor vasculature in the same specimen either visualized in optically cleared tissue volumes by i.v. injected α-PECAM-1 antibodies or in paraffin-embedded microtome sections by immunohistochemical staining (IHC) (scale bars: 100 µm). A mask (green) generated from the LSFM signal was overlaid onto the IHC staining to indicate the position of blood vessels. (**d**) Colocalization of dUnaG-3ALFA protein expression and neoepitopes formed by the hypoxia marker pimonidazole in wholemount stainings of a 1 mm vibratome section. Comparatively low pimonidazole signals are highlighted by yellow arrowheads (scale bars: 200 µm).

Combining intravenous (i.v.) injection of direct-labelled α-PECAM-1 antibodies prior to euthanasia allowed us to correlate the intratumoral vascular network (Supplementary Video S1) with hypoxia-detection, based on the genetically encoded dUnaG-3ALFA reporter. Thus, we were able to visualize the entire tumor vessel bed and to correlate its structure with the hypoxia status of the adjacent tissue (Fig. 2a,b; Supplementary Video S2). We validated the LSFM-derived α-PECAM-1 signals by paraffin embedding (PE) of cleared tissue samples after LSFM, followed by alternating hematoxylin/eosin (H&E) or immunohistochemical PECAM-1 staining of 5 µm sections, using a different α-PECAM-1 antibody. For a rough correlation of sections and LSFM volumes, we aligned the H&E staining pattern with the tumor outline, already providing a good match of the larger vessels (Supplementary Figure S3). Having established the orientation of LSFM and PE tissue volumes, we were able to precisely match the vascular pattern in LSFM and α-PECAM-1 immunohistochemical staining, demonstrating the validity of the LSFM-based volumetric vessel assessment (Fig. 2c).

Representative thin planes taken from digitally rendered LSFM-derived image volumes illustrate enhanced dUnaG-3ALFA expression in poorly vascularized regions, distant to tumor blood vessels, suggestive of limited oxygen availability (Fig. 2b). Reliable detection of hypoxic tumor volumes by the UnaG-based hypoxia-reporter was confirmed, in agreement with a recent study^40^, by colocalization of neoepitopes formed by the bioreductive pimonidazole-derivative *Hypopxyprobe* and sites of UnaG-3ALFA expression (Fig. 2d and Supplementary Video S3). Taken together, a newly established wholemount staining protocol allowed nanobody-mediated detection of a genetically encoded hypoxia-reporter throughout complete murine intracranial gliomas.

### Analysis of large light sheet microscopic datasets using computational image-analysis pipelines

**Figure 3:**
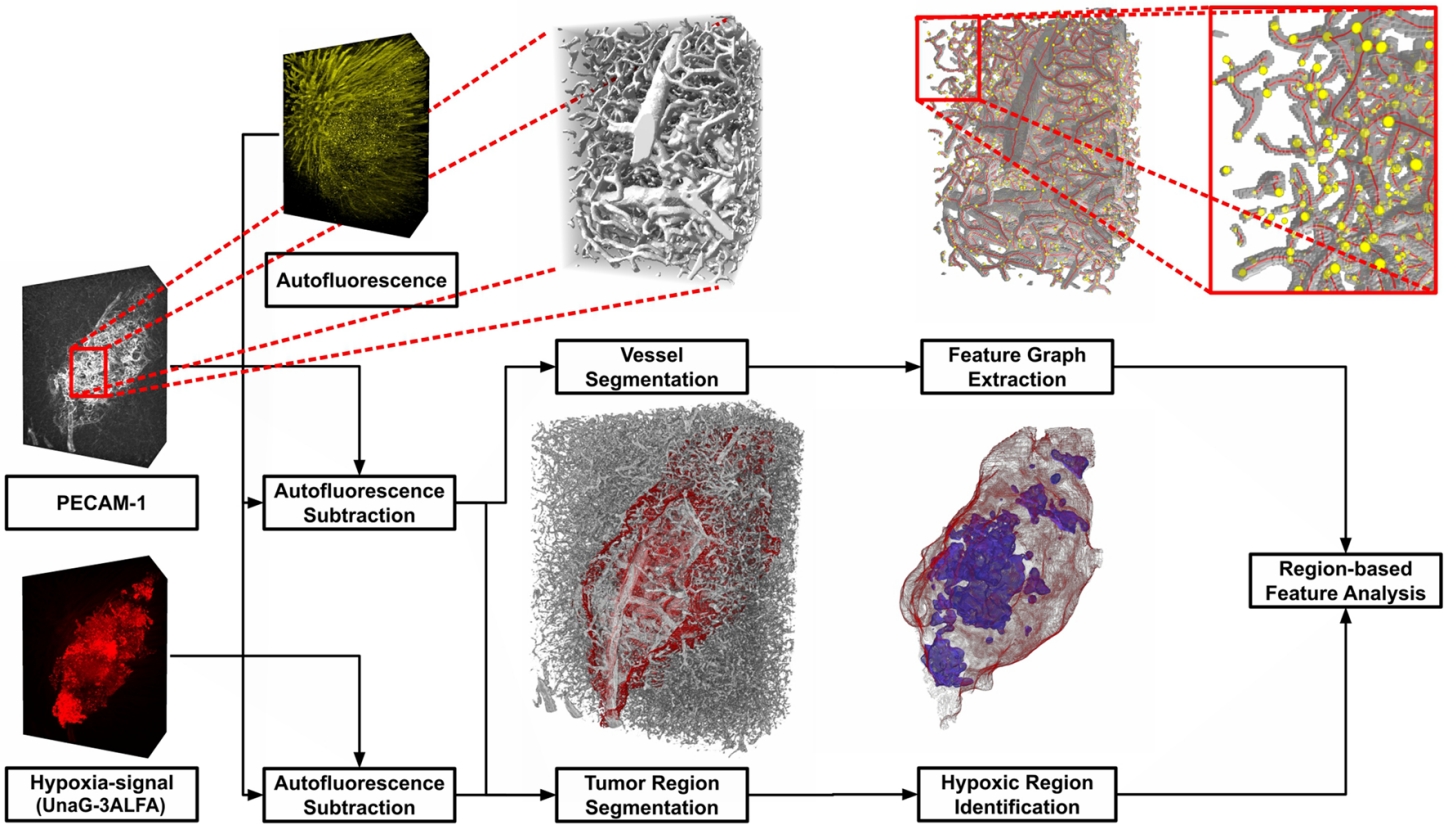
Overview of the pipeline for region-based feature analysis. For preprocessing, autofluorescence was subtracted from the PECAM-1 and UnaG-3ALFA channels, after intensity normalization. A full binary segmentation of the vascular volume, including the lumen, was derived from the PECAM-1 signal using a 3D-Unet based refined machine learning model. The graph representation and according features of the extracted vessel segments were implemented in Voreen. In parallel, both signals were used to delineate hypoxic tumor regions. Two coarse segmentations of the tumor volume were calculated using first, thresholding on the UnaG-ALFA signal and second, a simple 3D-Unet based segmentation model operating on the PECAM-1 signal. After combination, both segmentations were corrected manually. Hypoxic tumor regions were identified using intensity thresholding on a smoothed version of the UnaG-3ALFA signal. Finally based on their location, vessel segments and features were assigned to the resulting regions (healthy, normoxic and hypoxic) within the volume.

Next we addressed the impact of different treatment modalities on vessel structure and tumor oxygenation. Therefore, we xenografted 5 × 10^4^ Gli36 cells, stably transfected with HRE-dUnaG-3ALFA, intracranially into SCID mice and euthanized the animals after 21 days, as previous experimentation had established that these parameters would preclude development of a severe health burden in the control groups. Sample preparation and LSFM image acquisition followed stereotypically the workflow outlined in Supplementary Figure S4. Size and complexity of the tumor vasculature precluded manual annotation and required to develop an automated computational analysis (Fig. 3) to capture quantitative features in different sample regions. Our pipeline aims to accurately segment strongly varying vessel sizes, requiring (i) the elimination of false positive signals due to autofluorescence (AF) and (ii) a robust segmentation including the lumen of large vessels that lack a luminal PECAM-1 signal. Subsequently, (iii) geometric features have to be extracted from the vasculature segmentation and (iv) tumor volumes as well as contained hypoxic regions need to be identified.

### Vessel processing and segmentation

**Figure 4:**
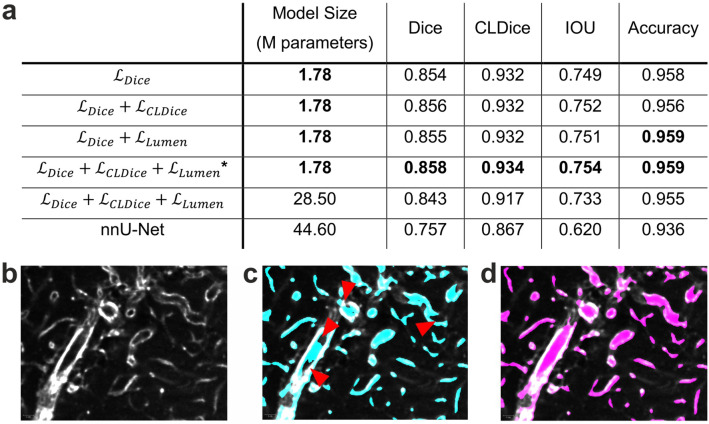
Qualitative and quantitative representation of the proposed vessel segmentation approach for lumen segmentation of large vessels. (**a**) Quantitative results: All metrics were calculated on binarized segmentations using a threshold of 0.5, with test data derived from image volumes not seen during training. The proposed method is marked with *, bold values indicate best performance per criterion. Results from nnU-Net are obtained using the official repository. All rows except the last are trained using a 3D-UNet with the training procedure described in the methods section. (**b**) Single optical plane depicting the intratumoral PECAM-1 signal. (**c**) Segmentation result of a model trained with both Dice loss and Centerline Dice loss. Missing lumen in the segmentation is indicated by red arrowheads. (**d**) Lumen segmentation after inclusion of the lumen loss.

Addressing (i), AF present in subregions of some image volumes was captured in the 525/50 nm channel (Supplementary Figure S5) and subtracted from both the dUnaG-3ALFA and PECAM-1 signal. Differences in overall absolute signal intensity and the presence of intensity gradients in the imaged volumes prohibited the use of a global subtraction method (Supplementary Figure S5). Instead, we took an approach based on the appearance of AF in all channels with the same relative spatial distribution albeit with different intensity. AF signal peaks were detected using the nearest neighborhood for comparison. After normalization of both AF and target signal, subtraction revealed previously hidden structures (Supplementary Figure S5). For (ii), the segmentation of the vascular network, a machine learning model was trained on manually annotated data to segment the vascular structure and was specially tuned to identify the lumen of large, irregular vessels (Supplementary Figure S6; Supplementary Video S4). To this end, we extended established state of the art methodology for segmentation of vessel-like structures^32^ with an additional lumen loss term, using the Euclidean Distance Transformation of the ground truth segmentation as a weight map to focus big vessels in the proposed loss. Specifically, a small 3D-Unet^41^ with approx. 1.7 million parameters was trained using a weighted combination of three loss terms, normal dice loss together with the clDice proposed by Shit et al.^32^ for accurate centerline prediction, as well as the new lumen-centered loss (for details see Methods Section Deep-Learning based vessel segmentation). Evaluation of the proposed method on unseen, manually annotated test data showed a clear improvement of the segmentation quality in comparison to off-the-shelf methods like nnU-Net^33^ (Fig. 4a), raising the dice score from 0.757 to 0.858. Albeit deploying the lumen loss does not lead to a substantial improvement in terms of standard metrics (Fig. 4a), in comparison to only using the dice and centerline dice loss, a better lumen reconstruction was observed for vessels with larger diameter (Fig. 4b–d). Increasing the size of the model by multiplying the number of channels in each convolution slightly decreased the segmentation quality, nevertheless, both architectures outperformed the established nnU-Net substantially (Fig. 4a). Further increasing the size of the network to approx. 45 million parameters yielded similar training losses, but overfitting could be observed based on the increasing test loss.

### Feature extraction and tumor region segmentation

After calculating the segmentation, a graph and feature extraction was performed using the algorithm by Drees et al.^29^ implemented in Voreen^42^, to address challenge (iii). The robust design and inherent scalability of this algorithm are ideally suited for the analysis of the large image volumes obtained by LSFM. The excerpt and zoom shown in the pipeline overview (Fig. 3, top right) demonstrate reliable graph construction for vessels of all scales. For each vertex in the graph representation, a set of features describing the corresponding vessel segment was calculated, allowing comparison of different sample regions based on their vessel segments and calculated feature distributions. Available features included the average cross section, radius, volume, minimum and maximum radius, as well as length and curveness of the segments.

**Figure 5:**
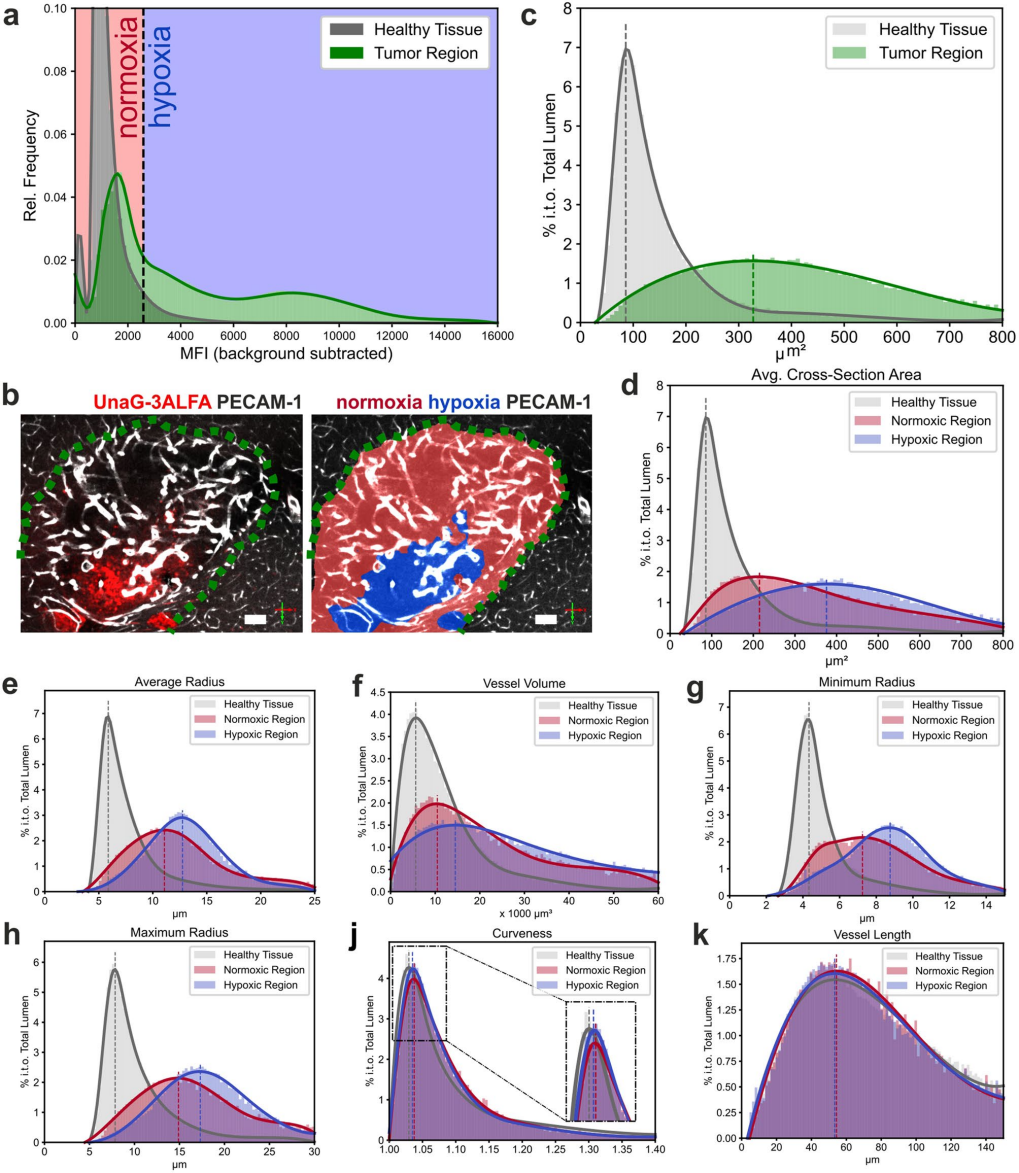
Analysis of the cerebral and tumor vascular architecture and classification of tumoral oxygenation patterns within vehicle-treated (PBS) mice. (**a**) Intensity distribution within dUnaG-3ALFA fluorescence channel. Based on the autofluorescence of the brain parenchyma (grey), a hypoxia threshold was defined at 1.5σ (black dotted line) above the mean. (**b**) Representation of tumor (delimited by green dotted line) oxygenation clusters in an optical sectional plane of an LSFM image stack (left panel: immunostaining of dUnaG-3ALFA-positive tumor regions (red); right panel: normoxic (low UnaG-3ALFA, red) and hypoxic (high UnaG-3ALFA, blue) tumor tissue; scale bars: 100 µm). (**c**) Distribution of the average cross section area (vessel volume / length) of cerebral (grey) and tumor (green) blood vessels in the segmented vessel beds (n = 4). (**d**–**k**) Analysis of architectural vascular features including average cross section area (**d**; volume / length), average radius (**e**), vessel volume (**f**), minimum radius (**g**) and maximum radius (**h**), curveness (**j**) and vessel length (**k**) in the surrounding cerebral parenchyma and normoxic and hypoxic oxygenation clusters of the tumor blood vessel bed (% in terms of (i.t.o.) total lumen; n = 4). Variations of the obtained vascular features are significant between all clusters (healthy tissue/normoxic region, healthy tissue/hypoxic region, normoxic/hypoxic region; Kolmogorov–Smirnov test; Supplementary Tables S1). Dotted lines represent maxima of the respective normal distributions (**a, c**–**k**).

For region-specific analysis, addressing (iv), tumors were segmented semi-automatically, by manual combination of two segmentations. Specifically, to capture the distinct vessel morphology of tumor regions, a 3D-UNet based segmentation model using a downscaled version of the PECAM-1 signal as an input was trained. A second, threshold-based segmentation calculated on the dUnaG-3ALFA signal was deployed, complementing the first segmentation. In contrast to the complex vasculature, tumor regions can easily be annotated such that manual inspection and corrections were combined with the segmentations when necessary, using VASTLite^43^. The resultant tumor volume was then clustered into normoxic and hypoxic regions via a threshold for the dUnaG-3ALFA specific signal intensity, which was set to 1.5σ of the mean AF intensity (MFI) of the brain parenchyma, individually considering the sample background (Fig. 5a). This approach accounts for normoxic background expression of the dUnaG-3ALFA mCMV-promoter by assigning low dUnaG-3ALFA expression to normoxia. Allocation of the resulting clusters to an optical section of an LSFM volumetric dataset verified the assignment of high dUnaG-3ALFA expression and the designated hypoxia-cluster (Fig. 5b; Supplementary Video S4).

### Extracted features reveal a distinct vascular morphology in normoxic and hypoxic tumor regions

The extracted vascular features allowed a robust discrimination of healthy brain parenchyma and tumor tissue (Fig. 5c). Of a range of parameters the average cross section area most robustly reported differences between hypoxic and normoxic tumor regions (Fig. 5d–k). The average cross section area was more than threefold increased in blood vessels of untreated tumors, reflecting the highly abnormal vessel architecture. To correlate features of the divergent vessel architecture to differently oxygenated tumor regions, we assigned them to the different tumor oxygenation clusters or healthy tissue and plotted the distribution of vessel cross section area, radius, volume, minimum radius and maximum radius (Fig. 5d–h), revealing differences of the vascular network in healthy parenchyma and normoxic or hypoxic regions of untreated tumors (PBS). Vessels with a smaller cross section area were more frequent within normoxic tumor regions (red; highest frequency: 200 µm^2^), whereas hypoxic tumor regions contained predominantly enlarged vessels (blue; highest frequency: 370 µm^2^) (Fig. 5d). Similarly, other vessel parameters based on luminal size like radius and volume distinguished normoxic and hypoxic tumor regions (Fig. 5e–h). Statistically significant shifts between the distributions of all extracted vascular features, including curveness and length, were revealed between all clusters of the vehicle-cohort (PBS) (Fig. 5d–k; Supplementary Table S1), healthy tissue/normoxic region, healthy tissue/hypoxic region, normoxic/hypoxic region), demonstrating the statistical power of the approach.

### Tumor volumes and oxygenation patterns are altered in response to systemic therapies

**Figure 6:**
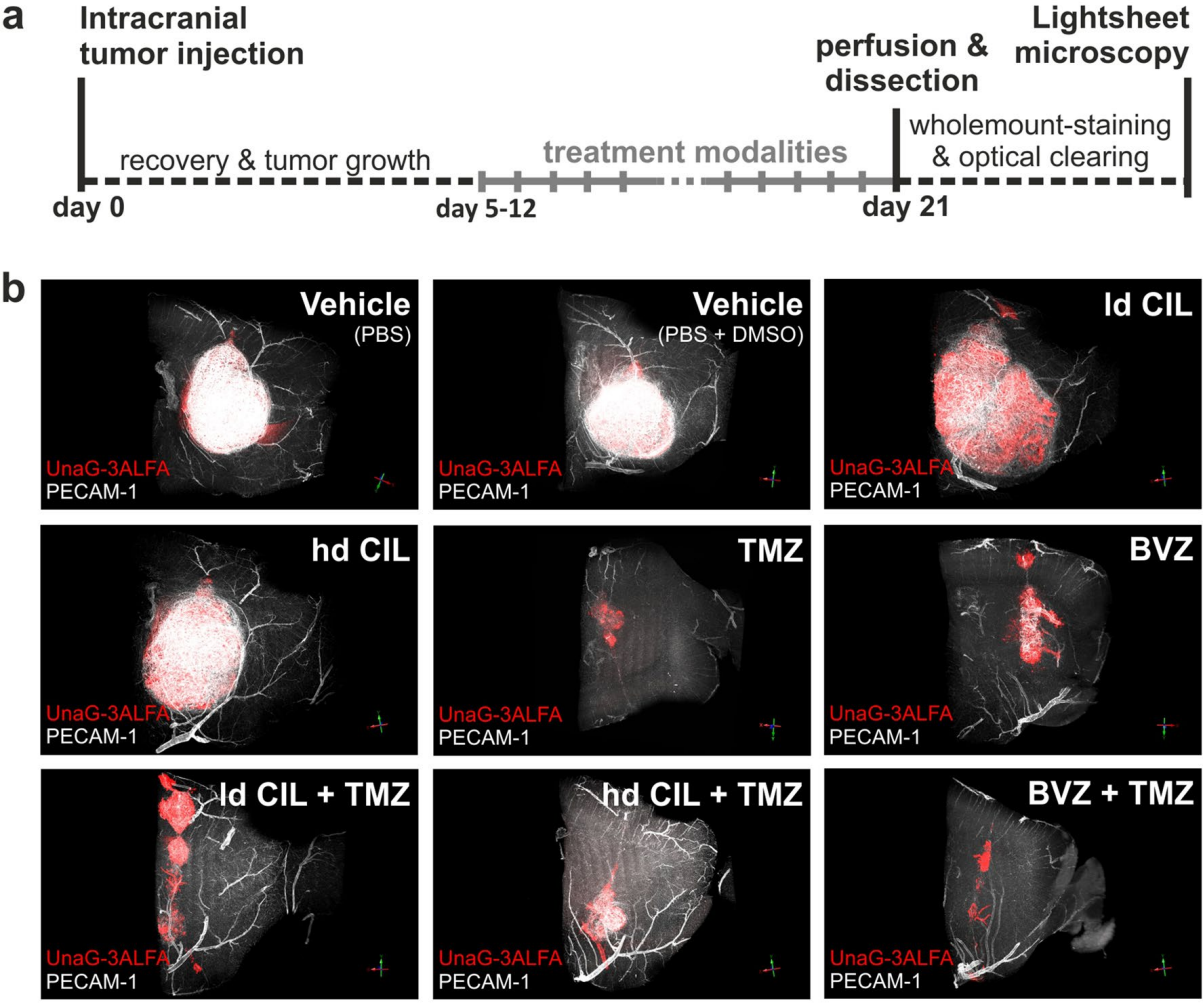
Assessment of the vascular architecture and hypoxia status in a murine glioblastoma model after different tumor therapies. (**a**) Between day 5 and 12 after intracranial injection of Gli36 cells, stably transfected with a construct for the hypoxia-inducible expression of dUnaG-3ALFA, SCID mice were treated intraperitoneally with temozolomide (TMZ) and/or low-dosed cilengitide (ld CIL), and/or high-dosed cilengitide (hd CIL) and/or bevacizumab (BVZ). Animals were euthanized 21 days later and tumor-bearing brains were dissected, immunostained and optically cleared for light sheet fluorescence microscopy. (**b**) Digital volume renderings of cerebral and tumor vessels (PECAM-1: white) and the hypoxic intratumoral regions (UnaG-3ALFA: red) were analyzed to reveal apparent alterations in tumor growth, hypoxia and the vascular network in response to treatment.

To obtain a comprehensive view of the vascular normalization brought about by AAT, we subjected our murine Gli36-tumor model to treatment using BVZ, ld or hd CIL and the corresponding combination therapies with TMZ (Fig. 6a). Therapeutic response, modulation of the tumor vascular architecture and determination of the oxygenation state were visualized for the entire tumors by LSFM (Fig. 6b; Supplementary Figure S7, S8) and features of the tumor vessel beds after treatment with TMZ, BVZ and CIL as well as the combination therapies (BVZ + TMZ, ld CIL + TMZ, hd CIL + TMZ) were compared to vehicle-treated tumors (Fig. 6b; Supplementary Figure S7, S8).

**Figure 7:**
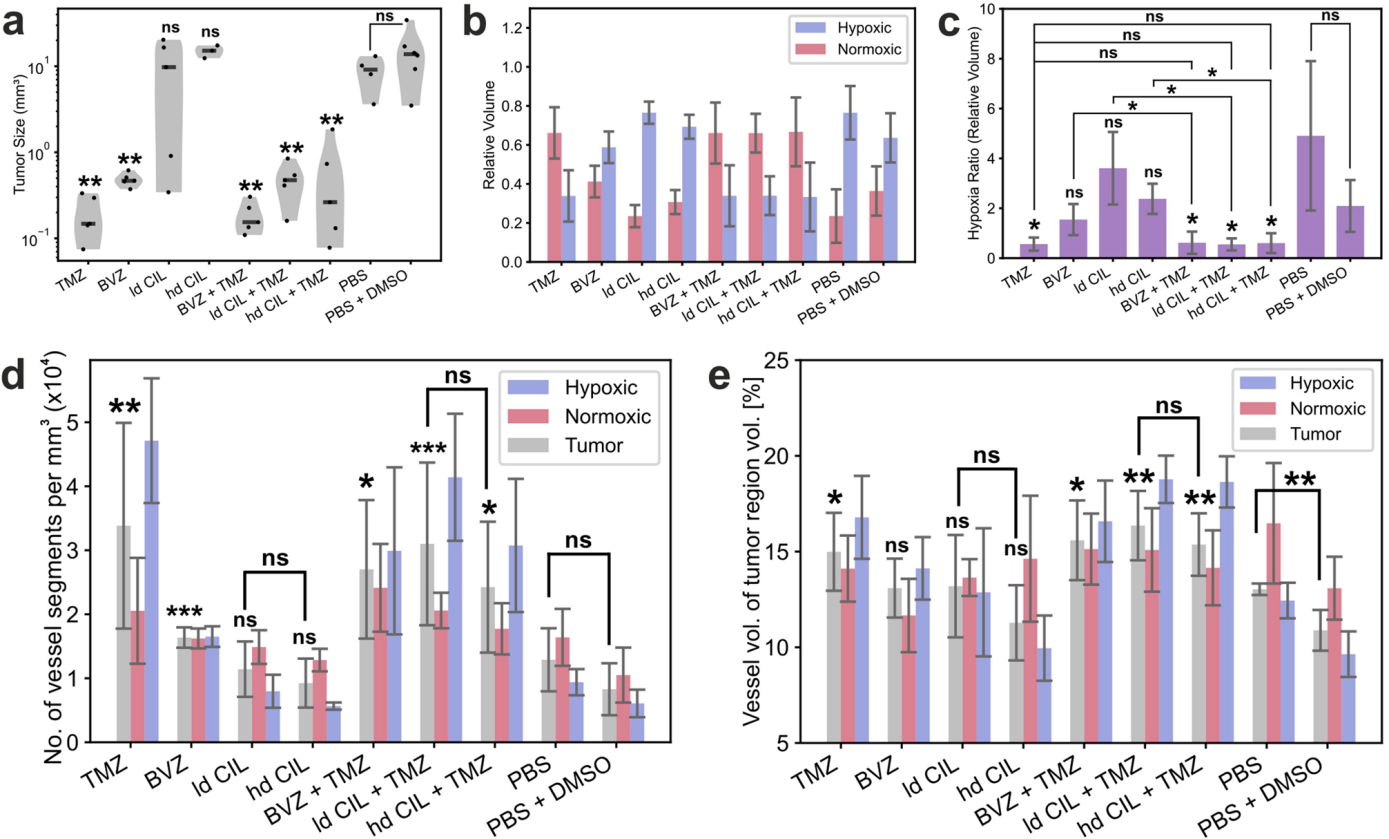
Tumor volumes and differential oxygenation patterns in Gli36 transplanted SCID mice after different therapies. Total volume and normoxic and hypoxic areas were identified in tumors treated with temozolomide (TMZ) and/or bevacizumab (BVZ), and/or low-dosed cilengitide (ld CIL) and/or high-dosed cilengitide (hd CIL). (**a**) Tumor volumes after the indicated treatment. (**b**) Relative hypoxic and normoxic tumor volumes and (**c**) hypoxia ratios (hypoxic/normoxic volume) in the indicated treatment groups. (**d**) Vascularization index as indicated by the number of vessel segments per mm^3^ in the tumor volumes and the contained oxygenation clusters. For statistical analysis, treatment groups were individually compared to vehicle controls (PBS, PBS + DMSO) if not indicated otherwise. (**a**, **c**, **d**; unpaired two-tailed Student’s t-test with Welch’s correction; *p* > 0.05, not significant (ns); *p ≤ 0.05; **p ≤ 0.01; ***p ≤ 0.001). Number of animals: TMZ: n = 5, BVZ: n = 5, ld CIL: n = 5 (**a**) or 4 (**b**–**d**), hd CIL: n = 3, BVZ + TMZ: n = 5, ld CIL + TMZ: n = 5, hd CIL + TMZ: n = 5, PBS: n = 4, PBS + DMSO: n = 6.

In agreement with a large body of preclinical studies, TMZ and BVZ monotherapy resulted in a highly significant reduction of tumor growth (Fig. 7a). TMZ showed the strongest effect, reducing the tumor volume 63-fold from 12 to 0.2 mm^3^, followed by a 24-fold reduction by BVZ from 12 to 0.5 mm^3^. Monotherapy with ld CIL or hd CIL was largely inefficient concerning tumor size (Fig. 7a), mirroring observations in other xenograft models and clinical trials, where tumor growth also was refractory to αvβ3-/αvβ5-integrin inhibitor monotherapy^15^. Combination of BVZ + TMZ showed no additivity and was most likely dominated by the effect of TMZ. Unexpectedly and irrespective of its dosage in combination therapy, CIL diminished the efficacy of TMZ in tumor growth reduction. In untreated tumors, the majority comprising approx. 2/3 of the tumor tissue was hypoxic (Fig. 7b,c), which remained largely unaltered after AAT monotherapy. Within the three AAT treatment groups, BVZ showed the lowest hypoxia/normoxia ratio, suggestive of a mild but insignificant improvement of hypoxia. In contrast, cytotoxic TMZ monotherapy, significantly alleviated hypoxia, indeed even reversed the hypoxia ratio, which surprisingly was also the case for all AAT combination therapies with TMZ (Fig. 7b,c and Supplementary Figure S7 a,e–g).

### Correlation of the modulation of tumor vascular architecture and oxygenation patterns in response to systemic anti-angiogenic and cytotoxic therapies

**Figure 8:**
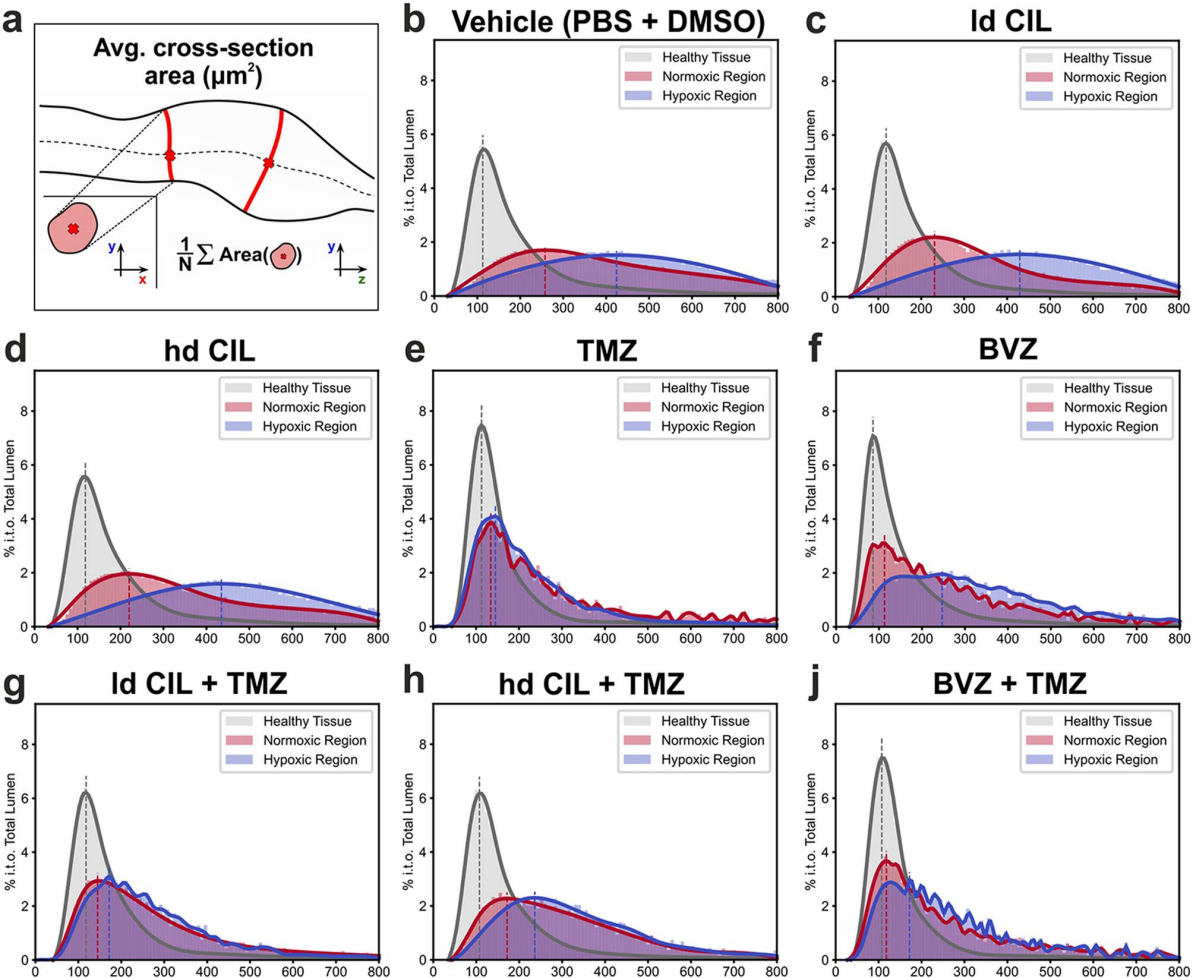
Specific changes of tumoral vessel architecture in relation to GBM oxygenation clusters (hypoxic and normoxic regions). (**a**) Quantification depicted as average cross sectional area (volume/length) between the treatment groups after systemic tumor treatments (**b**–**j**; % in terms of (i.t.o.) total lumen). Changes of average cross section area are significant between all clusters (healthy tissue/normoxic region, healthy tissue/hypoxic region, normoxic/hypoxic region) within each treatment group (Kolmogorov–Smirnov test; Supplementary Tables S1). Dotted lines represent maxima of the respective normal distributions. Number of animals: PBS + DMSO: n = 6, ld CIL: n = 4, hd CIL: n = 3, TMZ: n = 5, BVZ: n = 5, ld CIL + TMZ: n = 5, hd CIL + TMZ: n = 5, BVZ + TMZ: n = 5.

Assuming that the number of vessel segments per volume (mm^3^) provides a proxy for vascularization or vessel density, our quantitative feature extraction allowed the desired assessment of vessel regression after treatment, independently of the total tumor volume and oxygenation status (Fig. 7d,e and 8; Supplementary Figure S9). BVZ and TMZ monotherapy led to a significant increase in vessel segments per mm^3^, indeed arguing against vessel regression. Furthermore while the vessel volume per tumor volume was unchanged in the BVZ treatment group, the density of segments increased, suggesting an increase of smaller vessels, again supporting the concept of vessel stabilization (Fig. 7d,e). CIL monotherapy or had little to no effect on vascular density, irrespective of the treatment dose. In addition, combination therapy (CIL + TMZ) did not impact vessel density irrespectively of the CIL concentration (Fig. 7d,e). Like observed for the hypoxia ratio, TMZ appeared to dominate all combination treatments leading to an increase in vascular volume and density, which was in agreement with the overall reversion of the hypoxia ratio (Fig. 7b–e). Counterintuitively, we measured the highest vascular density in all TMZ treated tumors in dUnaG-3ALFA expressing tumor regions (Fig. 7d).

The robust differences detected in vehicle-treated tumors for average cross section area (Fig. 5d) prompted us to evaluate this parameter in all treatment groups (Fig. 8). In the control and the two CIL monotherapy groups, average vessel cross section area (Fig. 8a) distinctly differed between healthy parenchyma (grey), normoxic (red) and hypoxic (blue) tumor tissue (Fig. 8b–d; Supplementary Table S1). In hypoxic areas, large caliber vessels were particularly prominent, while in normoxic tissue the vessel cross section was about half-fold lower, but still more than twofold the value of healthy tissue (Fig. 8b–d). Treatment with TMZ reduced the vessel diameter in normoxic and hypoxic tumor tissue to values close to healthy tissue, although the differences in vascular morphology were still significant between parenchymal and tumoral vessels (Fig. 8e; Supplementary Table S1). BVZ treatment resulted in near vessel normalization in normoxic tumor tissue, while the vessels in hypoxic regions of the tumor had about the cross section area of vessels in normoxic parts of untreated tumors, i.e. about twice the area of healthy parenchyma (Fig. 8f). Combination therapies showed a vessel normalizing effect that was most likely due to TMZ, but did not fully reach the extent of TMZ monotherapy (Fig. 8g–j). Most importantly, although vessel cross section areas appeared normalized for some treatment groups, there remained a significant morphological difference between the tumor and parenchymal blood vessel architecture (Supplementary Table S1).

Taken together, in all treatment groups except TMZ monotherapy, tumor vessels with a reduced average cross section area (Fig. 8a), which should potentially favor perfusion and oxygenation, were predominantly localized within normoxic tumor regions (red). Surprisingly, the shift in vessel diameter had only a minor impact on the overall tissue oxygenation.

## Discussion

In this study, we applied LSFM-based volume imaging of complete, unsectioned tumors to quantitatively analyze the vascular changes caused by AAT, with the aim to unravel underlying mechanisms and consequences of AAT. The central question we wanted to address concerns the relative contribution of vessel normalization versus vessel regression to the effects caused by AAT. Furthermore, does vessel normalization result in improved perfusion, leading to a relief of tumor hypoxia? We addressed these questions using the established, orthotopic Gli36 glioma cell xenograft tumor model in conjunction with a genetically encoded hypoxia sensor, which allowed us to determine activation of the hypoxia response pathway as a proxy for tissue oxygenation. We applied the AATs BVZ, ld CIL and hd CIL either alone or in combination with the cytoxtoxic drug TMZ, while TMZ monotherapy provided a baseline for the activity of chemotherapy. Surprisingly, we found solid indication for vascular normalization in all treatment groups, most pronounced for TMZ monotherapy, while none of the treatment groups, including all AATs regimen, provided significant evidence for vascular regression.

The comprehensive quantitative analysis presented here is based on the consequent use of 3D volume data to evaluate the vessel bed in entire tumors. This requires efficient wholemount staining, which is frequently obstructed by high tumor tissue density, limiting antibody penetration. We dealt with this obstacle by developing an improved wholemount staining protocol, which includes an additional hyperhydration and permeabilization step in CUBIC-L^39^ reagent prior to immunostaining and detection of a triple ALFA-tag with a directly-labeled high affinity nanobody. This protocol provides improved permeabilization for the enhancement of nanobody penetration, but retains mild staining conditions compatible with a large host of antibodies and nanobodies and ensures optimal tissue transparency after clearing. We adapted our computational pipeline specifically for the segmentation of large diameter vessels that were frequently present within tumor tissue. This combination of wet-lab and computational advances enabled us for the first time to visualize the complete vessel bed and hypoxia status of the microenvironment of entire tumors treated with different modalities with cellular resolution. An immediate advantage of the approach is the high statistical power gained from the evaluation LSFM imaging-based volume data, where each image stack correspond to thousands of traditional histological sections. For instance, established features of tumor vessels, like increased tortuosity^44^ and enlarged diameter were robustly reflected in our data sets. The later was best represented by the average cross section area that markedly distinguished healthy brain parenchyma from tumor tissue, but intratumorally also the normoxic and hypoxic tumor clusters.

The alkylating cytostatic drug TMZ either alone or in combination with BVZ showed the strongest effect on tumor growth, also clearly surpassing the growth inhibition by achieved BVZ monotherapy. CIL as monotherapy was ineffective, while concomitant with TMZ, tumor growth was inhibited. Surprisingly however, CIL diminished the capacity of TMZ to inhibit tumor growth. In all treatment groups, growth inhibition was accompanied by a reduction in vessel average cross section area, strongly suggestive of vessel normalization. This was also the case for the CIL combination groups. Indeed in the ld CIL + TMZ group, the reduction of the average cross section area was comparable to the BVZ + TMZ group, suggesting that an effect other than simple sensitization to VEGF-A must be responsible for the reduced growth inhibition in these groups. In addition, TMZ mono and combination therapy resulted in a significant increase in the number of vessel segments per mm^3^ tissue, and concordantly a reduction of the hypoxic over normoxic tumor area ratio. Unexpectedly in the same groups, we noted the highest vessel segment density in the hypoxic tumor regions, potentially indicating reactive vascularization. Reasons could involve poorly functional vessels or development of escape mutants with unphysiological HIF-stabilization during the treatment regime, which would impair validity of the hypoxia clustering.

Taken together, while our analysis provided clear evidence for vessel normalization in all investigated therapy modalities that resulted in effective inhibition of tumor growth, we detected no indication of vessel regression, in particular not in the BVZ treatment group. Instead, after TMZ mono- and combination therapy with BVZ, curveness of tumor vessels approached the values of vessels from the surrounding parenchyma, further supporting the notion of normalized vascular structure in response to these therapies. Clearly, these statements are based on a single tumor model and require analysis of a variety of xenograft models, ideally with cell lines freshly established from patient material to derive more universally applicable insights. Also, our hypoxia clustering is based on activation of the physiological hypoxia response-pathway, which could potentially become impaired if tumor cells developed mechanisms of HIF1-stabilization independent of hypoxia. Nevertheless, our study demonstrates feasibility of a quantitative analysis of the vessel bed and microenvironment of entire tumors, paving the way to a better understanding of treatment modalities, in particular AAT.

## Methods

### Plasmids

For generation of the hypoxia-sensor construct HRE-dUnaG-3ALFA, a triple ALFA-epitope tag^38^ was inserted C-terminally of the UnaG cDNA into HRE-dUnaG^36^. For constitutive expression of dUnaG-3ALFA, the hypoxia-responsive promotor element in HRE-dUnaG-3ALFA, comprised of five hypoxia-responsive elements (HREs) fused to a minimal human cytomegalovirus (mCMV)-promoter (Addgene #46,926)^45^, was replaced by a CMV-promoter element resulting in CMV-dUnaG-3ALFA (Supplementary Figure S1). The functional sensor protein is destabilized (dUnaG-3ALFA) by a PEST-degradation motif, derived from the murine ornithine carboxylase gene^46^.

### Generation of stably hypoxia-expressing tumor cell lines

Human glioma Gli36 cells (RRID:CVCL_RL88)^47^ were obtained from Andreas Jacobs, EIMI, University of Münster. Cells were transfected with the HRE-dUnaG-3ALFA or CMV-dUnaG-3ALFA plasmids by calcium phosphate co-precipitation. For isolation of stable transfectants, cells were selected in the presence of geneticin (1 mg/ml G418) for 2 weeks. Following culture under hypoxia (1% ambient O_2_) cell bulks with intermediate expression level were isolated by FACS.

### Intracranial tumor induction^48^

Gli36 cell lines stably expressing dUnaG-3ALFA were intracranially injected into SCID mice (CB17/lcr-*Prkdc*^*scid*^/lcrlcoCrl (Charles River Laboratories, Wilmington, USA), 20–23 weeks, female). After anesthetization with carprofen and midazolam (intraperitoneal (i.p.)), anesthesia was maintained by inhalation of 1.5% isoflurane (O_2_ flow ~ 0.8 L/min). The shaved head was stereotactically fixed and the skull cap exposed. After orienting at bregma and moving the dental drill 0.5 mm rostral and 2 mm to the right, a hole was introduced into the calvaria of the right hemisphere. Using a micro-syringe, 5 × 10^4^ cells of a single cell suspension were injected 3 mm deep into the cortex, the hole was sealed with bone wax and the scalp closed with 3–4 interrupted stitches.

### Treatment modalities

Mice were administered with eight doses with either 10 mg/kg Bevacizumab (BVZ)^49^, 50 μg/kg (ld CIL)^16,50^ or 5 mg/kg Cilengitide (hd CIL)^16^ via i.p. injection every second day starting from day 5 after tumor implantation. For combination treatment, additional three doses of 10 mg/kg Temozolomide (TMZ)^51^ in DMSO were administered on day 13, 15 and 17. For single therapy, three doses of 10 mg/kg TMZ were administered on day 12, 14 and 16. Vehicle control mice were injected with 150 µl of PBS every second day, starting from day 5 after tumor implantation (PBS) or additionally with 25 µl of DMSO on day 13, 15 and 17 (PBS + DMSO). The transplanted cell number and the stereotypic experimental endpoint, 21 days after GBM xenotransplantation, were defined by the maximal time, control animals survived without developing a severe health burden. Health status was monitored by daily scoring of parameters including body weight, fur condition, breathing, bodily discharge, behavioral and neurological deficits or loss of coordination.

### Wholemount staining of intracranial tumors

After i.v. injection of Alexa Fluor™ 647-labelled rat monoclonal α-mouse PECAM-1 antibody (clone 5D2.6 and 1G5.1^52^), mice were perfused with isotonic saline solution followed by 4% (wt/vol) paraformaldehyde (PFA)/PBS under deep anesthesia. Brains were dissected and postfixed in 4% PFA for 2 h at room temperature (RT). Subsequently, hemispheres were cut sagittally and further reduced to the tumor containing volume (rostral right hemisphere, Fig. 1b). Specimen were washed in 1X PBS at RT (approx. 12 h), before transfer into CUBIC-L (10% (wt/vol) *N*-butyldiethanolamine, 10% (wt/vol) Triton X-100 in H_2_O)^39^ for delipidation and decolorization. Incubation in CUBIC-L lasted for one week at 37 °C while gently shaking and refreshing the reagent daily. After washing in 1X PBS for 24 h at RT, samples were placed in PermBlock solution (3% (wt/vol) bovine serum albumin, 0.1% (vol/vol) Tween20 in PBS) and further incubated at RT for 3 days. The ATTO 542-labelled recombinant single-domain antibody FluoTag®-X2 α-ALFA (NanoTag Biotechnologies GmbH, Göttingen, GER) was diluted in PermBlock (1:500) and added to the tumor bearing specimen for 3 weeks, while gently shaking at 37 °C in the dark. Final washing was performed in 1X PBS-T (0.1% (vol/vol) Tween20 in PBS) for 3 days with multiple changes of the medium.

### Immunohistochemical analysis of paraffin-embedded tissue samples after optical clearing

The optically cleared tissue samples were immersed in anhydrous methanol (2 × 99.8% (vol/vol) for 3 h at RT) to remove the BABB clearing solution after LSFM acquisition. Specimen were then processed to paraffin-embedded tissue blocks and immunohistochemistry (α-PECAM-1 (diluted 1:1000), ab124432, Abcam plc., Cambridge, UK) was performed on microtome sections. Paraffin sections were imaged with a Nikon Ti2-E epifluorescence microscope (Nikon BV, Amsterdam, NL), 20 × Plan Fluor objective (NA = 0.45) and a color camera (Nikon DS-Ri2). For image analysis Nikon NIS–Elements and FIJI by ImageJ^53^ were used.

### Hypoxia detection via pimonidazole

For bioreductive labelling of hypoxic tissue areas, tumor bearing mice were i.v. injected with 60 mg/kg bodyweight pimonidazole (Hypoxyprobe™-1), diluted in isotonic saline. After 30 min, animals were perfused. To counterstain intracellular pimonidazole-adducts, wholemount staining of 1 mm vibratome sections or an intact BVZ-treated tumor xenograft was performed via an ATTO 594-labelled rat monoclonal α-pimonidazole antibody (clone 11.23.22.R; Hypoxyprobe Inc., Burlington, USA). Optically cleared, stained tissue samples were visualized by LSFM.

### Optical tissue clearing of brain specimen^23^

The immunostained specimen were incubated in an ascending methanol series (50% (vol/vol) in H_2_O, 70% (vol/vol) in H_2_O, 2 × anhydrous (99.8% (vol/vol))), allowing 6 h for each step. Subsequently, the dehydrated samples were transferred to an anhydrous mixture of equal volumes of MeOH and BABB (1:2 (vol/vol) benzyl alcohol : benzyl benzoate). After complete equilibration, indicated by loss of buoyancy, the sample was finally moved into BABB.

### Light sheet microscopy

Optically cleared brain specimen were imaged using a LaVision UltraMicroscope II with Super Plan configuration and a 4 × objective (NA 0.35) (Milteny Biotec, Bergisch Gladbach, GER) equipped with a NKT supercontinuum white light laser (SuperK EXTREME FIU-15; NKT Photonics, Birkerød, DEN) and band-pass excitation filters (470/40 nm (AF), 546/10 nm (ATTO 542), 640/20 nm (Alexa Fluor™ 647). Acquisitions were performed using mono- or bidirectional illumination and dynamic focus positioning while applying a z-step size of 3 µm and a digital magnification of 0.6X. For emission detection, band-pass filters (525/50 nm (AF), 577/25 nm (ATTO 542), 690/50 nm (Alexa Fluor™ 647) were used and signals recorded with a sCMOS sensor camera (Excelitas PCO, Kelheim, GER).

### Preprocessing of reconstructed volumes

Image stacks were rendered using the *Voreen* (volume rendering engine) open-source volume visualization library and development platform (https://www.uni-muenster.de/Voreen/). After volume reconstruction, 3D images were digitally trimmed to the tumor-containing region and its immediate surrounding. For subsequent removal of AF, we evaluated the AF channel (525/50 nm) assuming a constant ratio of the relative AF intensity ratio in all channels. Peaks in the 525/50 nm band were detected using a neighborhood-based approach, specifically, the local average signal intensity was calculated by convolving the volume with a constant kernel of radius k (k = 32 in all conducted experiments). This local approach was necessary due to the inhomogeneity of the overall signal intensity throughout a volume. Using the obtained local average, signal intensities higher than 0.05 – 0.4 standard deviations (value chosen individually for every volume) above average were considered peaks to subtract. Due to the different intensity regimes of the AF and target signal, the peak signal was normalized by subtracting the (local) mean and dividing by the standard deviation first, and was then rescaled to fit the target signal by multiplying with its own standard deviation. In cases with more severe AF, the peak signal was amplified by factor of 1.2 before subtraction. During this stage, any bigger regions containing extremely high AF overpowering the target signal caused by effects like haemorrhages are masked for exclusion from the analysis at later stages.

### Deep-Learning based vessel segmentation

#### Data annotation

The segmentation model was trained based on a subset of the acquired volumes as training data, using only the PECAM-1 channel as input. In total, 4 volumes from different treatment modalities were used. Despite the low number of specimens, given the extensive size of each acquired image volume, a full manual annotation of the training set was infeasible. Instead, a pre-segmentation was obtained using classical methods (see below) and subsequently manually corrected and extended using VASTLite^43^ as annotation tool. For the initial segmentation, the image volume was first normalized based on the local average intensity, using the convolution operation from the AF preprocessing step. Then, hysteresis thresholding was used to create a segmentation, which subsequently got checked and corrected manually, with a particular focus on filling the lumen of bigger vessels.

#### Training details

For segmentation of the vascular network, we trained a basic 3D-UNet^41^ architecture on chunks of 1283 voxels. For inference, chunks were created with 50% overlap in all dimensions and stitched back together using Hann window stitching^54^. The four annotated volumes yielded 1705 chunks for training and validation. Best hyperparameters were chosen using a 20% validation set sampled randomly from all available chunks and the final model was trained using all available data. The training loss comprised three terms, specifically the normal dice loss^55^ together with the centerline dice loss introduced by Shit et al.^32^ and a novel loss term focusing on segmenting the lumen of bigger vessels. The latter is based on the Euclidean Distance Transform of the ground truth segmentation. Each voxel was assigned the closest distance to any background voxel, resulting in higher values inside bigger vessels, down to zero in the background region. To ensure equal weighting for the innermost lumen, this distance was clipped to a given maximum radius r and then normalized using a shifted sigmoid function. Experimental evaluations of the radius r yielded good results for r = 10 in our setting. The shifted sigmoid function was calculated as

$$\sigma_{{{\text{shifted}}}} \left( {x,\;r} \right) = \sigma \left( {x - \left( {r - 5} \right)} \right),{ }\;\sigma \left( x \right) = \frac{1}{{1 + e^{ - x} }}$$

with $\upsigma \left(5\right)\approx 1$. To calculate the loss, both predicted and ground truth segmentation were weighted using the obtained distance map, and normal dice loss was applied afterwards. Altogether, the total loss was calculated as

$$\mathcal{L}=\left(1-\upbeta \right)\left(\left(1-\mathrm{\alpha }\right)\cdot {\mathcal{L}}_{{\mathcal{D}}ice}+\mathrm{\alpha }\cdot {\mathcal{L}}_{{{c}{l}} {{{\mathcal{D}}ice}}}\right)+\upbeta \cdot {\mathcal{L}}_{{\mathcal{L}}{\fancyscript{umen}}}$$

with $\mathrm{\alpha }=\upbeta = 0.5$ for the final model. The standard 3D-UNet architecture with 8 channels in the first layer, doubling after every down-sampling step for a total of 5 hierarchy levels, was trained for 150 epochs with an initial learning rate of 0.0001 after 5 epochs of warmup, applying an exponential learning rate decay of 0.99 after every epoch. For data augmentation, random contrast change and additive gaussian noise were used, as well as applying a random bias field creating a global intensity gradient for 75% of training samples, randomly chosen. As post-processing, the probabilistic segmentation maps were threshold using a confidence of 0.5 and finalized by morphological hole filling to clear small gaps in the segmentation.

### Feature graph extraction

The obtained segmentation volumes were rescaled to isotropic resolution by super-sampling in the z-dimension before being further processed. Voreen’s^42^ implementation of Drees et al.’s algorithm for graph and feature extraction^29^ was used to create a graph representation of the vessels. As a result, vessel networks were extracted with nodes corresponding to branching points in the vascular network, connected by vessel segments represented as vertices. For each segment, a set of features describing the morphology of the vessel were calculated. In order to correctly identify abnormally deformed vessels in tumoral regions as contiguous, the bulge size parameter was set to 1 allowing for relatively large bulges.

### Tumor region segmentation and hypoxic region identification

To separate the tumoral region from the surrounding healthy tissue, a combination of two segmentation methods was applied. First, a basic 3D-UNet based machine learning model was trained to identify tumor regions based solely on the morphology of vessels using the PECAM-1 signal as input. Training data for this model was acquired from five manually annotated sample volumes by chunking them into cubes, analogous to the proposed vessel segmentation pipeline, with the addition of an upstream downsampling by a factor of two. This doubled the effective receptive field without increasing the required computational resources. In total, 406 cubes were used for training. Training parameters including epochs and learning rate were kept the same, using a standard binar cross entropy loss. Due to misclassifications for samples with small tumors, especially for samples with combination therapies, a second segmentation based on thresholding the average dUnaG-3ALFA signal expression level was introduced. Manually, a threshold delineating the higher background signal intensity in the tumoral region in comparison to the healthy tissue was determined. Both segmentations were then superimposed and manually corrected using VASTLite. To extract the hypoxic regions inside the tumor, a threshold based on the mean fluorescence intensity (MFI) histogram is deployed. Specifically, using the obtained tumor region segmentation, the threshold is set to 1.5σ above the mean intensity of the healthy tissue MFI. Since the overall signal intensity distribution for the healthy tissue is more stable, this results in a comparable yet sample-specific threshold for delineating normoxic from hypoxic regions.

### Region-based analysis

Each vessel segment identified by the graph extraction algorithm was assigned to its corresponding region that is healthy tissue, normoxic or hypoxic tumor region, based on the spatial location of both vertices in the graph. Segments with end points on the border of the processed image volume were excluded. Finally, feature distributions are plotted as histograms. To account for the different sized vessel segments, the histograms do not show relatively frequency, which would have biased the distribution towards smaller vessels. Instead, each vessel segment was weighted according to its lumen (measured as the sum of all voxels allocated to this segment) in relation to the total volume (short: % in terms of (i.t.o.) total lumen) occupied by the vascular network.

### Statistical analysis

Statistical significances of datasets presented in Fig. 7 were calculated by unpaired two-tailed Student’s *t*-test with Welch’s correction due to unequal sample sizes and/or the assumption of unequal variances, using GraphPad Prism software. Statistical analysis of the distributions of the extracted vascular features (Figs. 5 and 8; Supplementary Figure S9) was performed by nonparametric Kolmogorov–Smirnov test, calculating with 64-bit precision. Results of the latter are summarized in Supplementary Tables S1, pooling samples from the same treatment group. For each feature, regions of interest (healthy tissue, normoxic tumor, hypoxic tumor) were compared pairwise. A 95% confidence level (*p* < 0.05) was set, representing significances by asterisks: *p* > 0.05, not significant (ns); *p ≤ 0.05; **p ≤ 0.01; ***p ≤ 0.001 (NEJM-formatted).

### Approval for animal experiments

All procedures, scoring and monitoring involving animals were carried out in strict accordance with the German Animal Welfare Act (TierSchG). Procedures were reviewed by an anonymous ethics committee and approval was granted by the LANUV, the Federal State Agency for Nature, Environment and Consumer Protection of North Rhine-Westphalia (Recklinghausen) and is documented in the animal license TVA 81–02.04.2023.A019 (Münster). We comply with the ARRIVE guidelines.

### Supplementary Information

Supplementary Information 1.

Supplementary Video 1.

Supplementary Video 2.

Supplementary Video 3.

Supplementary Video 4.

## Data Availability

All data is available in the main text, the supplementary information or will be provided on demand. Code and an exemplary LSFM volume will be publicly available at https://zivgitlab.uni-muenster.de/cvmls/brain_tumor_analysis.
