# Supplementary material for: Therapy-induced modulation of tumor vasculature and oxygenation in a murine glioblastoma model quantified by deep learning-based feature extraction
Source: Sci Rep. 2024 Jan 23;14:2034. doi: 10.1038/s41598-024-52268-0 (PMC10805754; doi:10.1038/s41598-024-52268-0)
Supplement: Supplementary file 1 — Supplementary Information 1. [file 41598_2024_52268_MOESM1_ESM.pdf]

Therapy-induced modulation of tumor vasculature and oxygenation in a murine glioblastoma model quantified by deep learning-based feature extraction

Nadine Bauer[ł,1,2], Daniel Beckmann[ł,3,4], Dirk Reinhardt[1], Nicole Frost[1], Stefanie Bobe[1,5], Raghu Erapaneedi[1,2], Benjamin Risse[3,4], Friedemann Kiefer[1,2,*]

ł*shared first authorship*

[1]*European Institute for Molecular Imaging (EIMI), Multiscale Imaging Centre (MIC), University of Münster, Röntgenstr. 16, 48149 Münster, Germany*

[2]*Max Planck Institute for Molecular Biomedicine, Röntgenstr. 20, 48149 Münster, Germany*

[3]*Institute for Geoinformatics, University of Münster, Heisenbergstr. 2, 48149 Münster, Germany*

[4]*Institute for Computer Science, University of Münster, Einsteinstraße 62, 48149 Münster, Germany*

[5]*Gerhard Domagk Institute of Pathology, University Hospital Münster, Domagkstr. 15, 48149 Münster, Germany*

*corresponding author

Supplementary Information

1. Legends to Supplementary Videos
2. Supplementary Figures
3. Supplementary Tables
4. References

1. Legends to Supplementary Videos

Supplementary Video S1

Digital 3D-reconstruction of the cortical and tumor vessel beds in an intracerebrally xenografted human Gli36 tumor constitutively expressing UnaG imaged by light sheet fluorescence microscopy (LSFM). Following intravenous (i.v.) injection of fluorophore-labelled PECAM-1 antibody[1] for visualization of the cerebral and intratumoral vasculature (white), the tissue was dissected and fixed. Subsequently, the specimen was optically cleared and UnaG-fluorescence lost due to protein denaturation. After imaging by LSFM, visualization and 3D reconstruction of the volumetric microscopy data was performed using the open-source volume visualization library Voreen[2].

Supplementary Video S2

Intracranially xenografted HRE-dUnaG-3ALFA expressing Gli36 tumor cells reveal intratumoral hypoxia. To contrast cerebral and tumor vasculature, PECAM-1 antibodies[1] (white) were i.v. injected before sacrificing the animal. After permeabilization and delipidation in CUBIC-L[3], hypoxia-sensor expression was visualized by wholemount immunostaining with ALFA-tag[4] specific nanobodies (red) followed by optical clearing and LSFM. The open source framework Voreen[2] was utilized for visualization and 3D-reconstruction of the volumetric LSFM dataset.

Supplementary Video S3

Excellent colocalization of HRE-dUnaG-3ALFA-based (red) and pimonidazole-based (cyan) hypoxia signals in a xenograft tumor validated the tumor hypoxia indicated by dUnaG-3ALFA. 21 days after xenotransplantation, pimonidazole and PECAM-1 antibodies[1] were i.v. injected in BVZ-treated animals before preparation of the tumor-bearing hemispheres. After fixation and permeabilization, dUnaG-3ALFA and pimonidazole-adducts were immunostained by ATTO-labelled α-ALFA nanobodies (red) or α-pimonidazole antibodies (cyan) respectively, prior to optical clearing, LSFM imaging and 3D-reconstruction.

Supplementary Video S4

Digital 3D-reconstruction of the predicted vessel segmentation (white) and HRE-dUnaG-3ALFA fluorescence signal (red) of a vehicle-treated (PBS) Gli36 tumor. Despite considerable differences in morphology between tumoral and cranial vasculature, the vessel segmentation accurately reflects the vascular topology. In the second half of the video, the tumor volume is highlighted in blue and red, indicating regions assigned to either normoxic (red) or hypoxic (blue) oxygenation clusters, based on dUnaG-3ALFA expression levels. The threshold was calculated following the herein established method. Rendering and visualization was obtained using Voreen[2].

2. Supplementary Figures

Figure S1

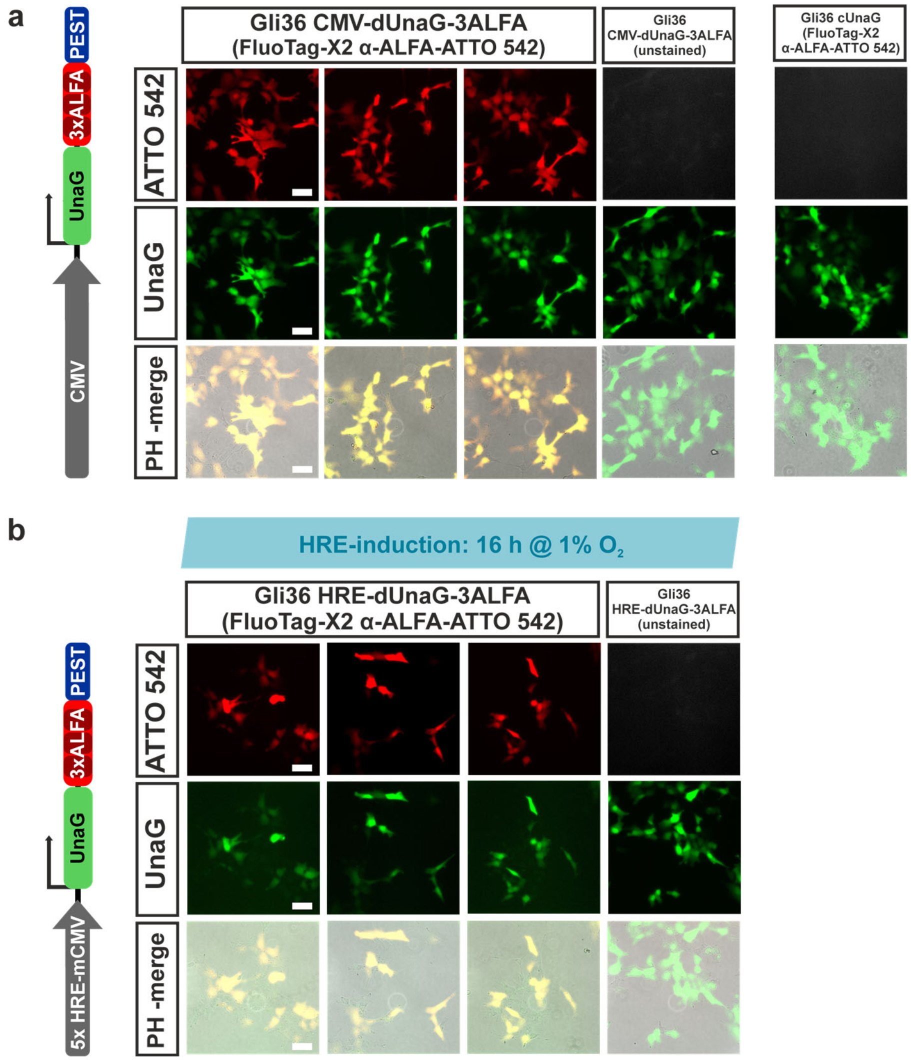

Nanobodies directed against the ALFA-tag specifically detect dUnaG-3ALFA in Gli36 cells. dUnaG-3ALFA sensor constructs were comprised of the UnaG[5] cDNA, followed by three copies of the ALFA-tag[4] (3xALFA) and a PEST-degradation motif[6]. Expression is either controlled by (**a**) a constitutive cytomegalovirus (CMV)-promoter or (**b**) by five hypoxia-responsive elements (HRE) fused to a minimal CMV (mCMV)-promoter[7] (hypoxia-inducible sensor).Transiently transfected Gli36 cells, were either incubated with ATTO 542-labelled α-ALFA nanobodies (NanoTag Biotechnologies GmbH, Göttingen, GER) (columns 1 – 3) or remained unstained (column 4). UnaG-based sensor expression was readily detected via the ALFA-epitope tag[4] (red) present in (**a**) CMV-dUnaG-3ALFA and (**b**) HRE-dUnaG-3ALFA (subsequent to hypoxia-incubation at 1% O_2), where the ATTO 542 signal co-localized with UnaG endogenous fluorescence (green). No signal was detected in the ATTO 542-specific channel within unstained controls or the negative control (column 5; CMV-UnaG: UnaG cassette contains no ALFA-tags). PH: phase contrast, scale bars: 50 µm

Figure S2

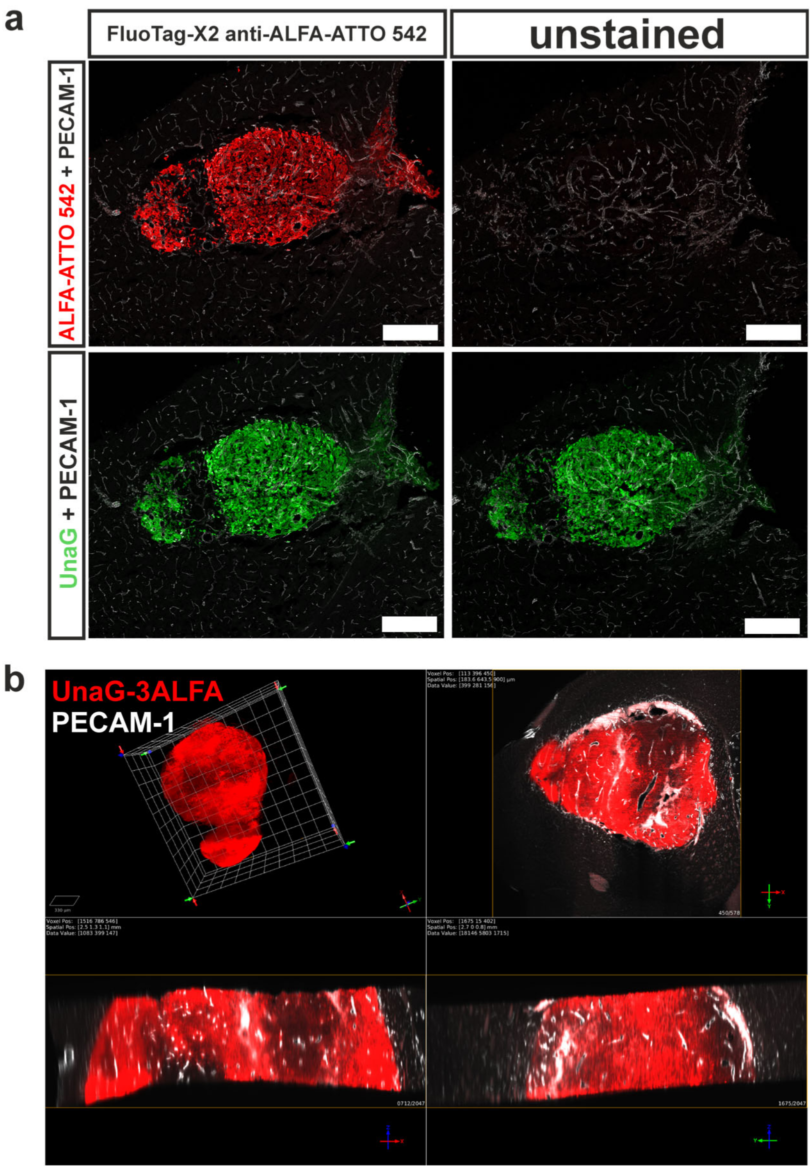

Staining of the entire volume of xenografted Gli36 tumors by α-ALFA nanobodies. Gli36 cells stably transfected with CMV-dUnaG3ALFA were intracranially xenotransplanted and tumors allowed to develop. After 21 days, animals were injected fluorophore-labelled PECAM-1 antibody[1] and subsequently sacrificed. Brains were dissected and wholemount immunostained with ATTO 542-labelled α-ALFA nanobodies (NanoTag Biotechnologies GmbH, Göttingen, GER). (**a**) In cryosections, the green fluorescence of UnaG and the ATTO 542 signal of the ALFA-tag (red) co-localized (laser scanning confocal microscopy; PECAM-1: white; scale bars: 300 µm). (**b**) Penetration efficiency of the ALFA-targetting nanobodies (red) was further assessed in 1 mm thick vibratome sections, which were optically cleared after immunostaining to allow visualization by light sheet fluorescence microscopy (PECAM-1: white).

Figure S3

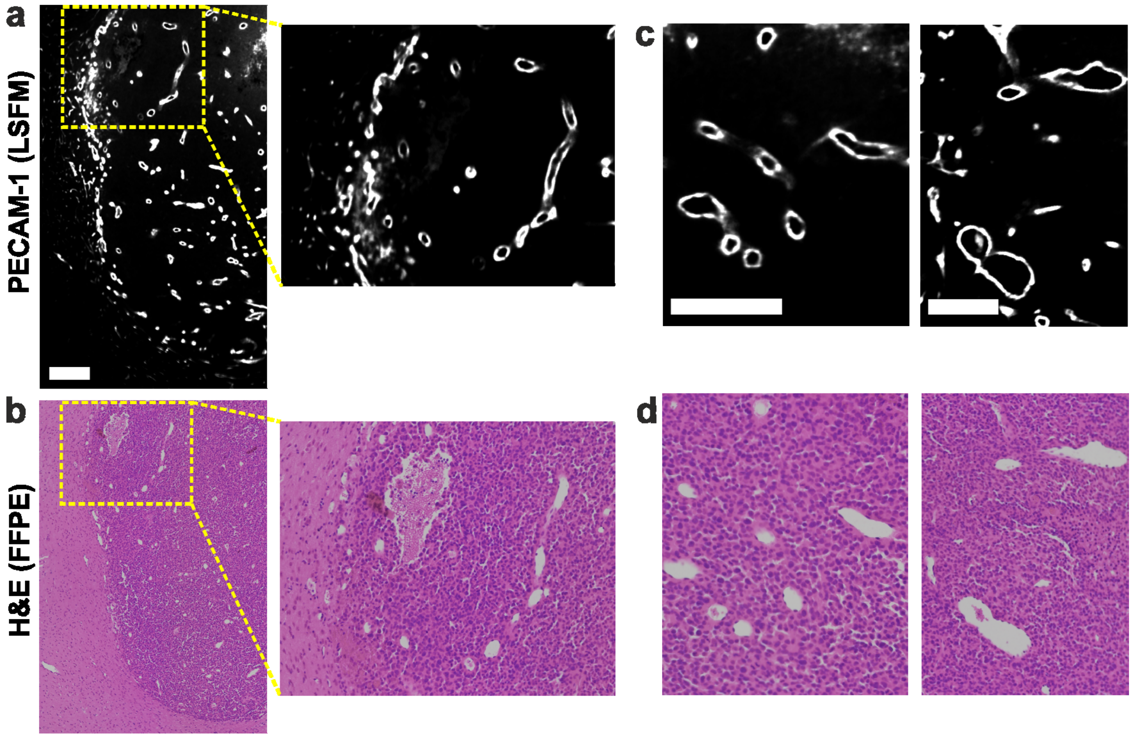

Correlation of α-PECAM-1 immunofluorescence staining in an optically cleared tumor volume imaged by LSFM and hematoxylin/eosin (H&E) staining of thin sections derived from the same sample after paraffin embedding. The pattern of large vessels was used for spatial correlation of both data sets (scale bars: 200 µm**).**

Figure S4

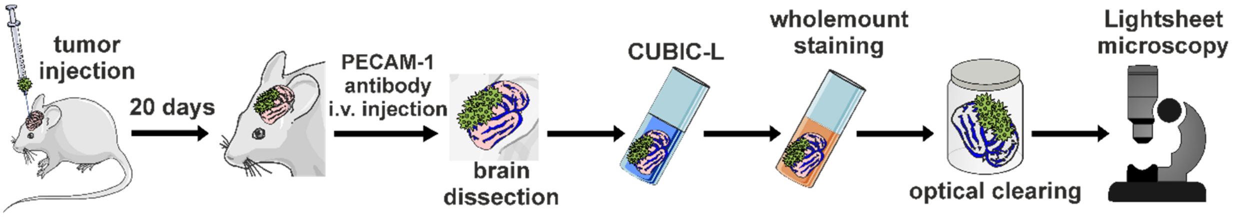

Work-flow of transplantation and sample processing for analysis of the vascular network and hypoxia status in glioblastoma xenotumors using LSFM. Gli36 cells (5 x 10^4) stably transfected with the hypoxia-sensor plasmid HRE-dUnaG-3ALFA were orthotopically, intracranially xenotransplanted into SCID-mice and tumors allowed to grow for 21 days. Subsequently to i.v. injection of fluorophore-labelled PECAM-1 antibodies[1] to contrast the vasculature, mice were perfused with isotonic saline solution followed by 4% paraformaldehyde (PFA)/PBS under deep anesthesia. After dissection of the brain, samples were processed into smaller tissue volumes, without affecting the tumor integrity and transferred into the hydrophilic CUBIC-L reagent[3] for permeabilization and delipidation. Prior to wholemount staining to detect hypoxia-induced dUnaG-3ALFA expression using ATTO 542-labelled α-ALFA nanobodies (NanoTag Biotechnologies GmbH, Göttingen, GER), unspecific epitopes were blocked using PermBlock buffer. Samples were extensively washed before proceeding with tissue dehydration and optical clearing in BABB in preparation for LSFM.

Figure S5

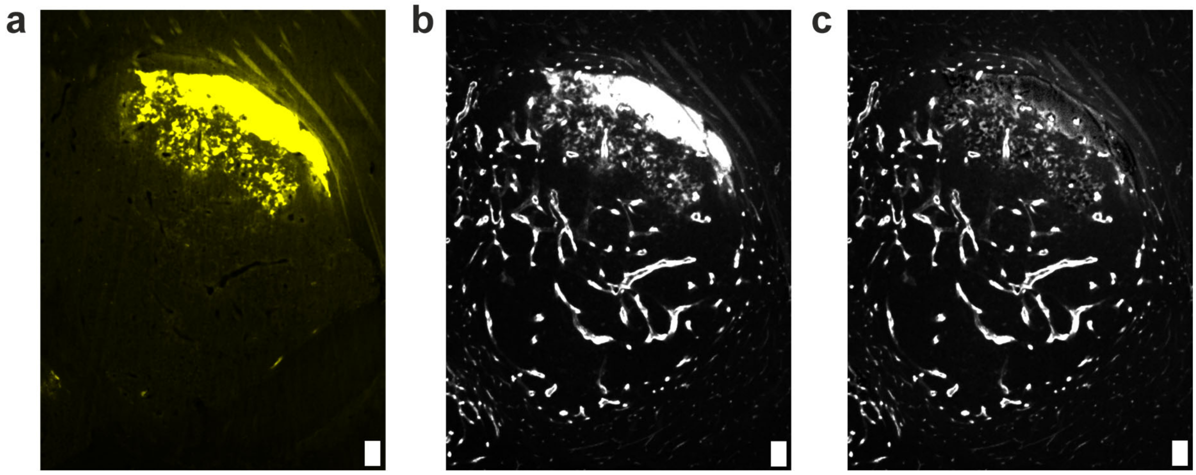

Subtraction of autofluorescent signals, shown for the PECAM-1 signal. (**a**) We used the green channel (525/50 nm) to detect autofluorescence. Regional peaks were detected as signal intensities exceeding the local, neighbourhood-based average intensity by a parameterized margin. To compensate for different absolute intensity regimes, both, autofluorescence and target signal, were normalized before subtraction. (**b**) Before subtraction, the PECAM-1 signal is overpowered by autofluorescence. (**c**) After subtraction, vascular structure within the highly autofluorescent region was restored. Scale bars: 80 µm.

Figure S6

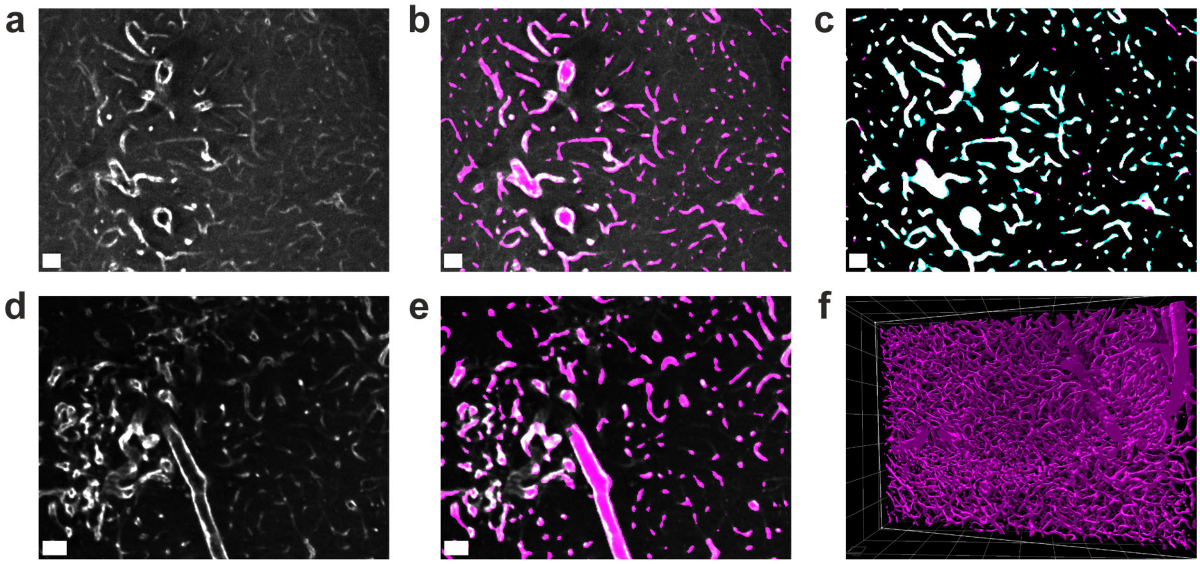

Vessel segmentation results on training data and unseen volumes. (**a**) Excerpt of a z-slice of PECAM-1 signal from a BVZ-treated volume used for model training. (**b**) The predicted segmentation (magenta), superimposed on the original signal, indicating good performance on training data. (**c**) Comparison of the predicted segmentation (magenta) with the annotated ground truth (cyan), resulting in white signal when overlapping with the predicted segmentation. (**d + e**) Analogous to (**a + b**), segmentation results are shown for unseen data from a TMZ-treated sample. The segmentation model generalizes well on smaller and bigger vessels for a wide range of the overall signal intensities, even on unseen data. Vascular topology is well preserved, even though some of the smaller vessels are underestimated. (**f**) A direct volumetric rendering of the predicted segmentation of the whole TMZ-treated sample highlights the complex topology of the vessel network. Scale bars: 50 µm.

Figure S7

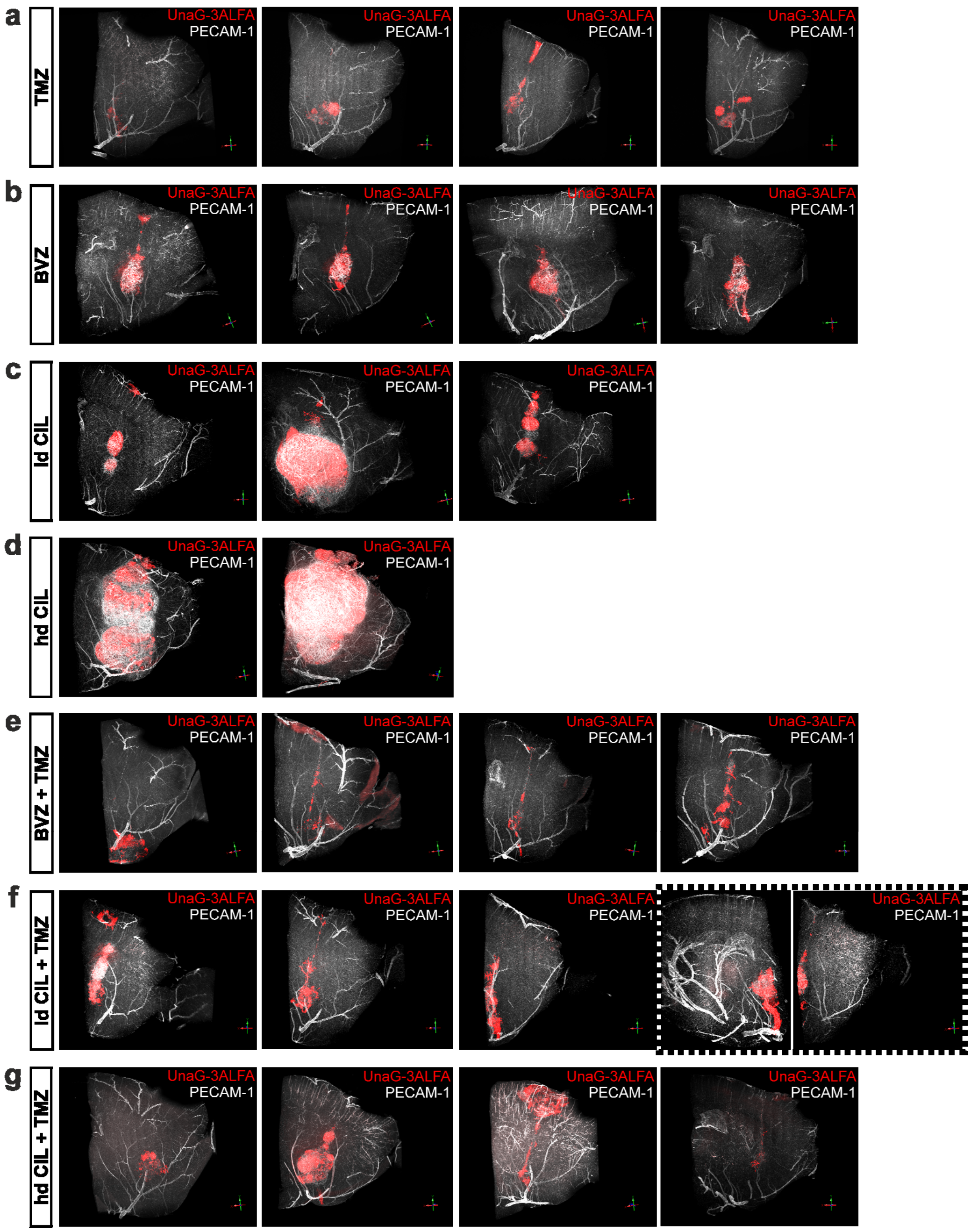

LSFM visualization of intracranial tumor hypoxia and vasculature after various treatment regimens. SCID-mice were intracranially, orthotopically xenotransplanted with human Gli36 cells stably expressing the hypoxia-responsive sensor dUnaG-3ALFA. The different treatment regimens were started 5 or 12 days after inoculation, respectively as follows: systemic monotherapies with (**a**) temozolomide (TMZ), (**b**) bevacizumab (BVZ), (**c**) low-dosed cilengitide (ld CIL) or (**d**) high-dosed cilengitide (hd CIL) and combination therapies with (**e**) BVZ + TMZ, (**f**) ld CIL + TMZ, (**g**) hd CIL + TMZ. Tumors were prepared 21 days after cell injection for LSFM and the cerebral and tumor vasculature (PECAM-1: white) as well as intratumoral hypoxia-sensor expression (UnaG-3ALFA, red) were visualized in 3D volumetric reconstructions.

Figure S8

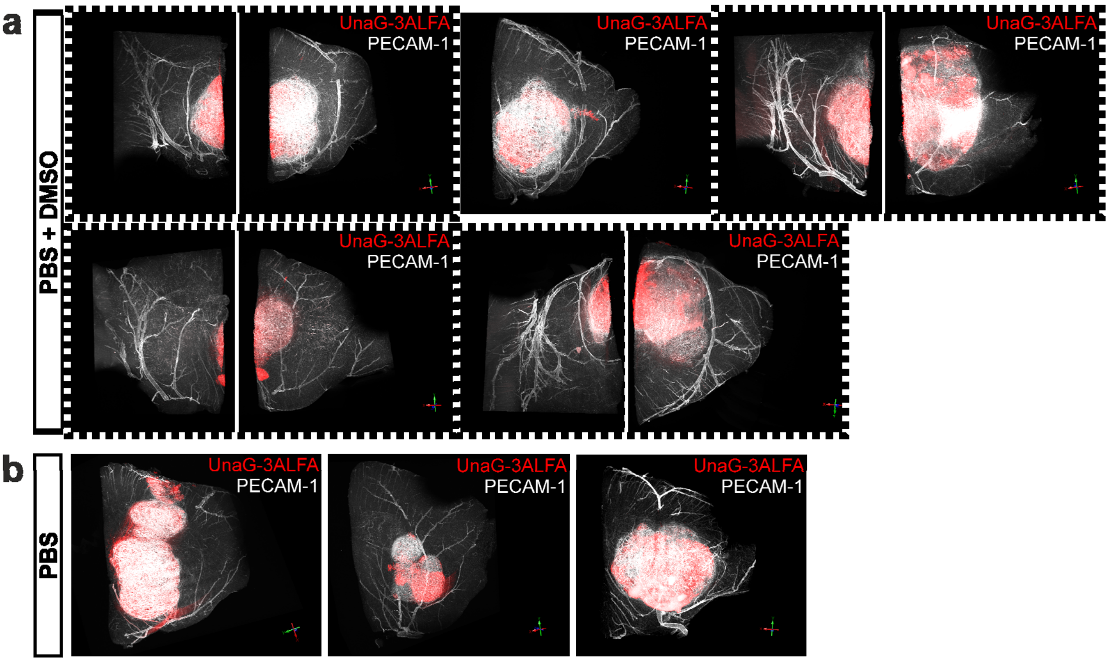

LSFM visualization of tumor hypoxia and vasculature in an orthotopic xenograft model of glioblastoma. Hypoxia and vasculature were visualized in tumors from vehicle-treated SCID-mice. After intracranial xenotransplantation of Gli36 cells stably expressing the hypoxia-responsive reporter protein dUnaG-3ALFA, mice were intraperitoneally injected with the vehicles (**a**) PBS + DMSO or (**b**) PBS, starting on day 5 after tumor cell injection. 3D visualizations revealed the vascular network (PECAM-1: white) and the hypoxic intratumoral regions (UnaG-3ALFA: red).

Figure S9

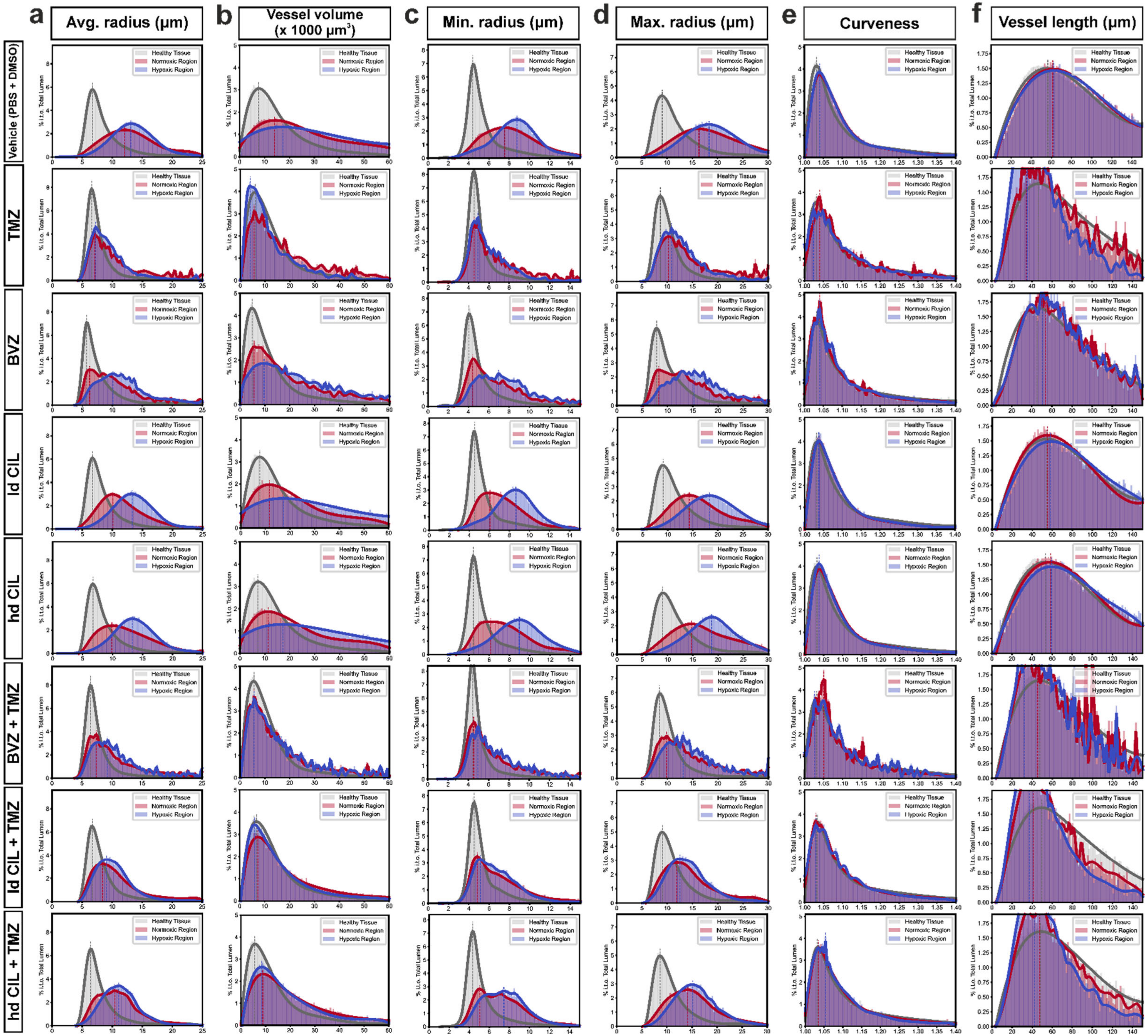
a Avg. radius (µm)
b Vessel volume (x 1000 µm³)
c Min. radius (µm)
d Max. radius (µm)
e Curveness
f Vessel length (µm)
Vehicle (PBS + DMSO)
TMZ
BVZ
ld CIL
hd CIL
BVZ + TMZ
ld CIL + TMZ
hd CIL + TMZ
Healthy Tissue
Normoxic Region
Hypoxic Region
% i.t.o. Total Lumen

Evaluation of the vascular structure in normoxic and hypoxic tumor volumes. The vascular features (**a**) average radius, (**b**) vessel volume, (**c**) minimal and (**d**) maximal radius, (**e**) curveness and (**f**) vessel length were extracted from volumetric datasets of adjacent parenchyma and intratumoral oxygenation clusters (hypoxic and normoxic regions) after applying the indicated systemic therapies. (**a - d**) Specifically the vessel radius and volume showed a distinctive correlation of vessel morphology and oxygenation status within the intratumoral oxygenation clusters. Particularly large vessels were present in hypoxic tumor volumes of the vehicle controls (PBS + DMSO) and the low-dosed or high dosed CIL-treated tumors when compared to other treatment groups. Although vascular modulation towards smaller vessels was observed in TMZ-treated mice and after combination therapy (BVZ / ld CIL + TMZ), vascular normalization did not seem to promote vascular perfusion and hence oxygenation as indicated by continued hypoxia-reporter expression, suggestive of hypoxic tumor clusters. (**e**) Vessel curveness appeared refractory to the different therapies, while we detected minor reductions in (**f**) vessel lengths for the TMZ, BVZ, BVZ + TMZ, ld CIL + TMZ and hd CIL + TMZ treatment groups. However, this feature strongly correlated with the overall reduction of the tumor volume and therefore was likely no consequence of their vascular modulating effect. Frequencies were represented and weighed in % in terms of (i.t.o.) total lumen. Number of animals: TMZ: n = 5, BVZ: n = 5, ld CIL: n = 4, hd CIL: n= 3, BVZ + TMZ: n = 5, ld CIL + TMZ: n = 5, hd CIL + TMZ: n = 5, PBS + DMSO: n = 6

3. Supplementary Tables

Supplementary Table S1

Statistical analysis of the distributions of the extracted vascular features between the resulting clusters of each treatment group. Application of the Kolmogorov-Smirnov test revealed significant changes between all clusters (healthy brain parenchyma, normoxic and hypoxic tumor regions) of all treatment groups for average cross section area, minimal, average and maximum radius (green). Few non-significant clusters were detected when comparing vessel length, curveness and volume (red). P values were calculated with 64-bit precision ($p > 0.05$, not significant (red); *$p \leq 0.05$; **$p \leq 0.01$ (yellow); ***$p \leq 0.001$ (green). Number of animals: PBS: n = 4, PBS + DMSO: n = 6, ld CIL: n = 4, hd CIL: n= 3, TMZ: n = 5, BVZ: n = 5, ld CIL + TMZ: n = 5, hd CIL + TMZ: n = 5, BVZ + TMZ: n = 5

Treatment group	Cluster a	Cluster b	Length	Curveness	Volume	Avg CrossSection	minRadius	avgRadius	maxRadius
**PBS**	healthy	normoxic	8,9089E-192	0	0	0	0	0	0
**PBS**	healthy	hypoxic	0	2,9246E-163	0	0	0	0	0
**PBS**	normoxic	hypoxic	7,22019E-51	2,1386E-151	0	0	0	0	0
**PBS + DMSO**	healthy	normoxic	2,5364E-162	0	0	0	0	0	0
**PBS + DMSO**	healthy	hypoxic	0	1,2353E-114	0	0	0	0	0
**PBS + DMSO**	normoxic	hypoxic	2,86117E-36	0	0	0	0	0	0
**TMZ**	healthy	normoxic	1,15642E-94	3,19732E-05	3,48452E-36	0	0	0	0
**TMZ**	healthy	hypoxic	5,471E-22	0,204111892	0,002387869	5,37605E-20	8,46661E-21	1,91472E-32	1,35444E-39
**TMZ**	normoxic	hypoxic	4,35949E-62	2,80905E-05	6,89383E-41	0	0	0	0
**BVZ**	healthy	normoxic	1,94817E-15	0,000321451	0	0	0	0	0
**BVZ**	healthy	hypoxic	0,001417393	0,000492361	1,81447E-72	7,0428E-206	7,8301E-176	5,9091E-192	6,0191E-188
**BVZ**	normoxic	hypoxic	8,14294E-22	1,74383E-06	2,2986E-233	0	0	0	0
**ld CIL**	healthy	normoxic	9,4174E-106	0	0	0	0	0	0
**ld CIL**	healthy	hypoxic	1,1993E-242	4,71983E-78	0	0	0	0	0
**ld CIL**	normoxic	hypoxic	7,20554E-46	7,4203E-233	0	0	0	0	0
**hd CIL**	healthy	normoxic	1,17073E-55	0	0	0	0	0	0
**hd CIL**	healthy	hypoxic	3,2846E-265	7,65407E-91	0	0	0	0	0
**hd CIL**	normoxic	hypoxic	1,56602E-60	5,3221E-227	0	0	0	0	0
**BVZ + TMZ**	healthy	normoxic	5,1258E-99	8,02443E-08	1,75666E-46	0	0	0	0
**BVZ + TMZ**	healthy	hypoxic	0,114304137	0,999873325	0,537134338	4,41296E-08	1,78495E-07	1,09338E-09	2,04878E-10
**BVZ + TMZ**	normoxic	hypoxic	1,28184E-93	2,0864E-07	3,7168E-44	0	0	0	0
**ld CIL + TMZ**	healthy	normoxic	0	7,30043E-58	6,04873E-72	0	0	0	0
**ld CIL + TMZ**	healthy	hypoxic	1,08691E-60	3,81202E-08	0,905238781	2,7183E-131	2,3074E-153	8,7223E-185	1,6706E-196
**ld CIL + TMZ**	normoxic	hypoxic	3,0592E-187	1,05654E-38	3,14616E-70	0	0	0	0
**hd CIL + TMZ**	healthy	normoxic	8,84718E-44	2,79943E-35	0	0	0	0	0
**hd CIL + TMZ**	healthy	hypoxic	2,12575E-28	7,77704E-10	1,31447E-60	0	0	0	0
**hd CIL + TMZ**	normoxic	hypoxic	2,16194E-16	2,1732E-20	0	0	0	0	0

4. References

1. Wegmann, F. *et al.* ESAM supports neutrophil extravasation, activation of Rho, and VEGF-induced vascular permeability. *Journal of Experimental Medicine* **203**, 1671–1677 (2006).
2. Meyer-Spradow, J., Ropinski, T., Mensmann, J. & Hinrichs, K. Voreen: A Rapid-Prototyping Environment for Ray-Casting-Based Volume Visualizations. *IEEE Computer Graphics and Applications* **29**, 6–13 (2009).
3. Tainaka, K. *et al.* Chemical Landscape for Tissue Clearing Based on Hydrophilic Reagents. *Cell Reports* **24**, 2196-2210.e9 (2018).
4. Götzke, H. *et al.* The ALFA-tag is a highly versatile tool for nanobody-based bioscience applications. *Nature Communications* **10**, 1–12 (2019).
5. Kumagai, A. *et al.* A Bilirubin-Inducible Fluorescent Protein from Eel Muscle. *Cell* **153**, 1602–1611 (2013).
6. Rogers, S., Wells, R. & Rechsteiner, M. Amino acid sequences common to rapidly degraded proteins: the PEST hypothesis. *Science* **224**, 1343–1346 (1986).
7. Vordermark, D., Shibata, T. & Brown, J. M. Green fluorescent protein is a suitable reporter of tumor hypoxia despite an oxygen requirement for chromophore formation. *Neoplasia (New York, N.Y.)* **3**, 527–34 (2001).
